# Functional rarity of plants in German hay meadows — Patterns on the species level and mismatches with community species richness

**DOI:** 10.1002/ece3.9375

**Published:** 2022-10-01

**Authors:** Gabriel Walther, Ute Jandt, Jens Kattge, Christine Römermann

**Affiliations:** ^1^ Friedrich Schiller University Jena Jena Germany; ^2^ German Centre for Integrative Biodiversity Research (iDiv) Halle‐Jena‐Leipzig Leipzig Germany; ^3^ Martin Luther University Halle Wittenberg Halle Germany; ^4^ Max Planck Institute for Biogeochemistry Jena Germany

**Keywords:** environmental conditions, European grasslands, functional rarity, mesic grasslands, species rarity, trait distinctiveness

## Abstract

Functional rarity (FR) — a feature combining a species' rarity with the distinctiveness of its traits — is a promising tool to better understand the ecological importance of rare species and consequently to protect functional diversity more efficiently. However, we lack a systematic understanding of FR on both the species level (which species are functionally rare and why) and the community level (how is FR associated with biodiversity and environmental conditions). Here, we quantify FR for 218 plant species from German hay meadows on a local, regional, and national scale by combining data from 6500 vegetation relevés and 15 ecologically relevant traits. We investigate the association between rarity and trait distinctiveness on different spatial scales via correlation measures and show which traits lead to low or high trait distinctiveness via distance‐based redundancy analysis. We test how species richness and FR are correlated, and use boosted regression trees to determine environmental conditions that are driving species richness and FR. On the local scale, only rare species showed high trait distinctiveness while on larger spatial scales rare and common species showed high trait distinctiveness. As infrequent trait attributes (e.g., legumes, low clonality) led to higher trait distinctiveness, we argue that functionally rare species are either specialists or transients. While specialists occupy a particular niche in hay meadows leading to lower rarity on larger spatial scales, transients display distinct but maladaptive traits resulting in high rarity across all spatial scales. More functionally rare species than expected by chance occurred in species‐poor communities indicating that they prefer environmental conditions differing from characteristic conditions of species‐rich hay meadows. Finally, we argue that functionally rare species are not necessarily relevant for nature conservation because many were transients from surrounding habitats. However, FR can facilitate our understanding of why species are rare in a habitat and under which conditions these species occur.

## Graphical abstract

Functional rarity represents a promising concept to better understand the ecological importance of rare species and may be a useful tool to protect functional diversity more efficiently. We investigate patterns and drivers of plant functional rarity in German hay meadows on both the species level (which species are functionally rare and why) and the community level (how is functional rarity associated with species richness and environmental conditions). Our results suggest that functional rarity is not necessarily relevant for nature conservation but can serve as a useful tool to better understand why species are rare in a habitat and under which conditions these species occur.

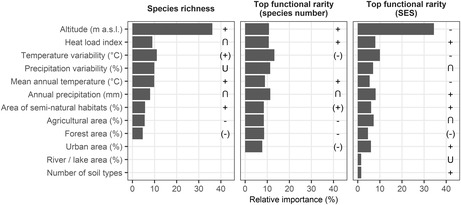

## 1 INTRODUCTION

Ecosystems typically consist of a few common and many rare species (Preston, 1948), each displaying a certain combination of traits, contributing to ecosystem functioning. During the last decades, many studies showed that functional diversity, i.e. “the value and range of functional traits of organisms present in a given ecosystem” (Diaz & Cabido, 2001), is key to understand how species in a community affect ecosystem functioning (Cadotte et al., 2011). Following the mass ratio hypothesis (Grime, 1998), it is assumed that ecosystem functioning is mainly driven by traits of the most abundant species. However, different studies show that rare species can also significantly contribute to ecosystem functioning by supporting ecosystem processes via indirect effects (Säterberg et al., 2019), ensuring ecosystem functioning via functional redundancy (Jain et al., 2014), or driving ecosystem processes via functionally unique trait attributes (e.g., keystone species; Marsh et al., 2000).

Although some rare species may disproportionally affect ecosystem processes (Marsh et al., 2000) it is still difficult to systematically identify these species and to assess the functional role of rare species in an ecosystem. To identify rare species that may disproportionately affect ecosystems, Violle, Thuiller, Mouquet, Munoz, Kraft, Cadotte, Livingstone and Mouillot (2017) provided a framework to quantify functional rarity (FR). As part of the concept of functional diversity, FR represents a feature of a species that combines the rarity of a species with its functional distinctiveness, given by its traits. On a local scale, e.g., a vegetation relevé, FR of a species can be quantified by calculating its rarity based on its local abundance and by measuring how different the species' trait attributes are compared to the co‐occurring species (trait distinctiveness). That way, species can be locally common or rare and simultaneously display either redundant or distinct trait attributes. On larger spatial scales, the size of a species' geographic range (restricted vs. widespread) in combination with its trait distinctiveness is used to define the species' FR (Grenié et al., 2017, 2018; Violle, Thuiller, Mouquet, Munoz, Kraft, Cadotte, Livingstone & Mouillot, 2017).

So far, only few studies investigated FR either focusing on patterns of FR on the species level or on spatial patterns of FR and its overlaps with species richness. More specifically, the first group of studies examines the relationship between rarity and trait distinctiveness on the species level to identify processes leading to the observed patterns. High trait distinctiveness is often expected to be displayed by rare species only (Chapman et al., 2018; Mouillot et al., 2013) with different processes shaping this relationship. For example, environmental filtering may select for an optimal trait set that maximizes species abundance (Maire et al., 2012; Umaña et al., 2015) leading to low trait distinctiveness and low rarity. However, functionally similar species suffer from competition, resulting in lower abundances (MacArthur & Levins, 1967; Mouillot et al., 2007), i.e., high rarity at low trait distinctiveness. To reduce competition, species may focus on a special but scarce resource whose use requires specialized traits, while resource availability simultaneously limits species abundance (i.e., high trait distinctiveness and high rarity; Chapman et al., 2018; Gaston, 1994). However, there are also studies reporting that both rare and common species support high trait distinctiveness. For example, Chapman et al. (2018) argued that common species as well must display distinct trait attributes to have an advantage in competition for resources. Ambiguous results may be due to differences between organism groups and a limited focus on only one spatial scale in most studies (but see Mouillot et al., 2013), although species rarity might differ depending on the spatial scale under consideration (Rabinowitz, 1981). Though we lack a consistent understanding of how trait distinctiveness and rarity are related on different spatial scales, these findings suggest that FR can be a useful tool to explain patterns of species rarity on different spatial scales.

The second group of studies investigates spatial patterns of FR to identify hotspots of FR and explain their occurrence in relation to species richness. For example, in two global analyses on the FR of fish species, Grenié et al. (2018) and Trindade‐Santos et al. (2022) show that species‐poor regions harbor more functionally rare species than expected by chance and vice versa. Since conservation strategies mainly focus on taxonomic diversity (Pollock et al., 2017), these results have major implications for nature conservation showing that we do not protect all facets of biodiversity (Grenié et al., 2018; Trindade‐Santos et al., 2022). In consequence, Grenié et al. (2018) suggested to use FR as an additional prioritization criterion in nature conservation. However, it is unclear if this pattern persists across other organism groups and on smaller spatial scales.

With up to 67 different vascular plant species per meter square (Klimeš et al., 2001), European grasslands are hotspots of plant biodiversity harboring many rare and threatened plant species (e.g., in Germany, approximately 40% of all threatened plant species occur in grasslands; BfN, 2014) and supporting important ecosystem functions, e.g., biomass production (Hector et al., 1999) and carbon storage (Lange et al., 2015). Semi‐natural grasslands have developed over centuries under different land use practices (e.g., mowing, grazing; Hejcman et al., 2013). Due to changes in land use over the last decades (i.e., intensification, abandonment, and conversion), diversity in grasslands is declining (Janeček et al., 2013). Apart from land use, studies show that plant species richness strongly depends on local climate and topography (Divíšek & Chytrý, 2018; Irl et al., 2015). However, models identifying and explaining hotspots of species richness in Central European grasslands are sparse (but see Divíšek & Chytrý, 2018).

So far, no study has systematically quantified FR of plant species for European grasslands or linked plant FR to species richness or environmental conditions. Therefore, large knowledge gaps remain considering the patterns and drivers of plant FR on both the species level (i.e., which species are functionally rare and why) and the community level (i.e., how is FR associated with biodiversity and environmental conditions). Closing these gaps is essential before deciding if plant FR should be considered in nature conservation strategies. It might be possible that a species that is functionally rare in a specific habitat might be functionally common in other habitats because trait distinctiveness and rarity may differ depending on the habitat type. In consequence, species identified as functionally rare might not be important for nature conservation per se. Still, FR may be a useful tool to better understand why some species are rare in a habitat and under which conditions these species occur.

In this study, we analyze the patterns and drivers of FR for plant species from German hay meadows by combining data from 6500 vegetation relevés and 15 ecologically relevant functional traits. We investigate how the concept of FR can be used to better understand which species are functionally rare in a certain habitat and why, and under which conditions these species occur, which can be seen as a basis for further nature conservation evaluations. On the species level, we examine how species rarity and trait distinctiveness as the two components of FR are associated with each other on different spatial scales and how the observed patters can be explained by functional traits. To better understand patterns of FR on the community level, we investigate the relationship between species richness and FR and model species richness and FR depending on environmental variables.

## 2 METHODS

### 2.1 Vegetation data

**FIGURE 1:**
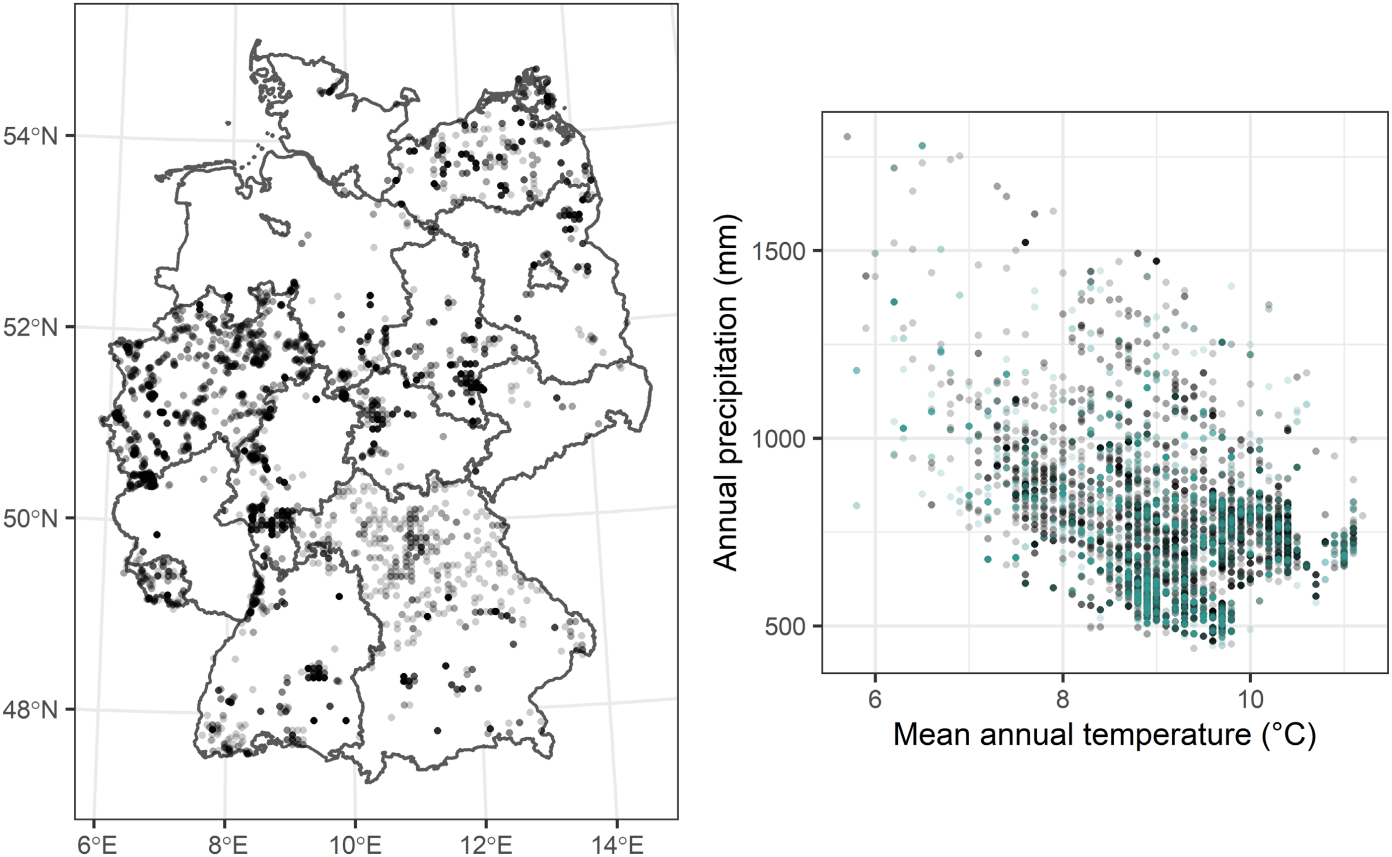
Distribution of 6500 selected hay meadows across Germany (left), and within climate space (right). Unselected relevés are shown in turquoise to illustrate even distribution in climate space. Darker colors indicate locations of higher relevé density. Climate data were derived from CHELSA (Karger et al., 2017, 2018).

Our study is based on vegetation data from the German Vegetation Reference Database (vegetation‐db.biologie.uni‐halle.de) providing 10,205 pre‐selected grassland relevés in Germany containing *Arrhenatherum elatius*, a grass species indicative for hay meadows. Based on species composition and abundance, relevés were assigned to different habitat types as defined by the EUNIS classification expert system (Chytrý et al., 2020) following Bruelheide et al. (2021). For our analysis only relevés that were classified as “Low and medium altitude hay meadows” were selected (hereafter referred to as “hay meadows” only). To ensure compatibility, species names in all data sources were standardized and aggregated to fit taxonomic concepts in the EUNIS expert system (Appendix S3 in Chytrý et al., 2020). Relevés without georeferences or with a location uncertainty of more than five kilometers were excluded. Although relevés were clumped in geographic space they were evenly distributed in climate space (Figure 1). Mosses and lichens were excluded from the further analysis. In addition, shrub and tree species were excluded since traits are available for adult individuals, whereas hay meadows are defined as meadows without adult trees and shrubs. Cover values of species occurring in multiple vegetation layers were summarized per relevé via

(1) $$1-\prod_{l=1}^{n}\left(1-p_{l}\right),$$

where *p*_*l*_ is the cover of a species in layer *l* and *n* the number of layers where this species occurs (Fischer, 2015).

From the selected 6500 relevés, 218 species that occurred in at least 1% of the selected relevés (corresponding to 65 relevés; approach adapted from Bruelheide, 2016) were included in further analyses. The application of the threshold excluded 611 species from the analysis of which only two were diagnostic for hay meadows (based on the list of 49 discriminating species for the selected habitat type in Appendix S3 of Chytrý et al., 2020). Hence, we argue that excluded species were likely randomly associated with the habitat at the time when the vegetation survey was done and do not represent rare hay meadow species sensu stricto (e.g., *Stellaria holostea*, *Viola arvensis*). Relevé size was not consistently available but mostly ranged between 10 and 100 m^2^.

### 2.2 Trait data

Table 1 gives an overview on the 15 used traits, including their ecological relevance. For continuous traits from TRY (Kattge et al., 2020), species mean values were calculated. Sparse data (i.e., less than three measurements per trait), were complemented by gap‐filled trait data (procedure is described in Schrodt et al., 2015). The other traits were derived from the respective source following the definition given in Table 1. Appendix S1 gives a detailed description of trait data cleaning and aggregation. Although intraspecific trait variability can affect ecosystem processes (Lecerf & Chauvet, 2008) and community assembly (Jung et al., 2010) our quantification of FR is constrained to the use of species mean trait values (Carmona et al., 2017; Grenié et al., 2017; but see Violle, Thuiller, Mouquet, Munoz, Kraft, Cadotte, Livingstone, Grenie & Mouillot, 2017). To account for redundancy of traits, only loosely correlated traits were used, i.e., *r*_S_ < .5 (Table S1). See Tables S2–S4 for the species‐trait matrix and a summary statistic of the traits.

**TABLE 1:** Overview of traits and their ecological relevance for grassland species

Trait [type (levels); unit]	Definition	Ecological relevance	Data source
Specific leaf area [continuous; mm^2^/mg]	Leaf area per leaf dry mass	Resource capture, usage, release (Diaz et al., 2004, 2016; Poorter et al., 2009; Westoby, 1998; Westoby et al., 2002; Wright et al., 2004)	Kattge et al. (2020)
Leaf dry matter content [continuous; g/g]	Leaf dry mass per leaf fresh mass	Resource capture, usage, release (Gross et al., 2007) Resistance to hazards (e.g. herbivory; Elger & Willby, 2003; Louault et al., 2005)	Kattge et al. (2020)
Leaf N per area [continuous; g/m^2^]	Leaf nitrogen content per leaf area	Photosynthetic capacity (Wright et al., 2004)	Kattge et al. (2020)
Plant height [continuous; m]	Vegetative plant height	Competition for light (Moles et al., 2009; Westoby, 1998; Westoby et al., 2002) Susceptibility to disturbance (e.g. mowing; Gross et al., 2007) Persistence over time (Westoby, 1998) Dispersal distance (Thomson et al., 2011) Reproduction (time to first reproduction, number of seeds per plant per year, size of seeds per plant per year; Moles et al., 2004, 2009)	Kattge et al. (2020)
Seed mass [continuous; mg]	Seed dry mass	Seedling survival (establishment success, tolerance to hazards during establishment; Moles & Westoby, 2006; Westoby, 1998; Westoby et al., 2002) Number of seeds (Moles & Westoby, 2006) Time to first reproduction (Moles & Westoby, 2006) Incorporation of seeds into soil (also depending on seed shape; Bekker et al., 1998)	Kattge et al. (2020)
Seed shape [continuous; dimensionless (0: spherical — 0.2: disk‐/needle‐like)]	Variance of seed length, width and thickness, each scaled by seed length (Thompson et al., 1993)	Incorporation of seeds into soil (also depending on seed mass; Bekker et al., 1998) Persistence of seeds in soil (Cerabolini et al., 2003)	Kattge et al. (2020)
Rooting depth [continuous; m]	Depth of roots in soil	Water and nutrient uptake (Comas et al., 2013; Maeght et al., 2013; Nippert & Holdo, 2015; Skinner & Comas, 2010) Anchorage (Comas et al., 2013)	Kattge et al. (2020) and Kutschera et al. (1982, 1992)
Flower duration [continuous]	Number of flowering months per year	Period of pollination (affecting sexual reproduction; Bock et al., 2014; Primack, 1985; Zhao et al., 2013)	Klotz et al. (2002)
Maximum lateral spread [ordinal (<0.01, 0.01–0.25, >0.25); m/year]	Maximum horizontal distance between parental and offspring plant	Short‐distance migration (Klimešová et al., 2018) Space occupancy after disturbance (Herben et al., 2018; Klimešová et al., 2018) Nutrient acquisition (Klimešová et al., 2018) Competitive ability (Klimešová et al., 2018)	Klimešová et al. (2017)
Maximum clonal multiplication rate [ordinal (<1; 1; 2–10; >10)]	Maximum number of offspring shoots per parental plant and year	Population size and persistence/space occupancy (Klimešová et al., 2019)	Klimešová et al. (2017)
Number of bud bank levels [continuous; 1–5 levels (<−10 cm; −10 to 0 cm; 0 cm; 0–10 cm; >10 cm)]	Number of levels where buds for vegetative regeneration are available	Regeneration after disturbance (Klimešová & Klimeš, 2007; Klimešová et al., 2018)	Klimešová et al. (2017)
Ratio between number of aboveground vs. belowground bud bank levels [continuous; dimensionless (−1: belowground only — 1: aboveground only)]	Fraction of aboveground bud bank levels minus fraction of belowground bud bank levels occupied	Disturbance avoidance (e.g. frost, fire, trampling, plowing; Klimešová & Klimeš, 2007; Klimešová et al., 2019)	Klimešová et al. (2017)
Number of clonal growth organs [continuous]	Possible number of different clonal growth organs	Probability of pursuing various clonal plant strategies (e.g. asexual reproduction, regeneration after disturbance, carbohydrate/nutrient storage, population persistence, spatial mobility; Klimešová et al., 2019; Van Groenendael et al., 1996)	Klimešová et al. (2017)
Mycorrhizal status [ordinal (facultative; obligate; non‐mycorrhizal)]	Mycorrhizal status based on the continuity of association with mycorrhizal fungi	Nitrogen/phosphorus acquisition (van der Heijden et al., 2015)	Guerrero‐Ramírez et al. (2020)
Legume [categorical; yes/no]	Taxonomic affiliation with Fabaceae	Nitrogen acquisition via microbial association with rhizobia (Long, 1989)	Kattge et al. (2020)

*Note*: References for data sets from TRY (Kattge et al., 2020) are listed in Appendix S3.

### 2.3 Environmental data

Table 2 gives an overview of all parameters used to describe environmental conditions per relevé. As a proxy for drought stress, we combined latitude, slope, and aspect to calculate heat load following Equation (3) in McCune and Keon (2002). Around each relevé location a circular buffer area with a radius of 927 m was used to calculate the value of different environmental variables, corresponding to the maximum edge length of the coarsest raster of environmental data (Table 2). For climate variables and variables derived from the digital elevation model, the mean of all raster values was calculated, weighted by their relative area within the buffer zone. For soil data, the number of different soil types within the buffer area was counted. Land cover classes were aggregated at higher levels (Table S5) and their proportional area within the buffer zone was calculated.

**TABLE 2:** Overview of environmental variables used to model species richness, number of functionally rare species, and its standardized effect size per relevé

	Min.	Mean	Max.	Data type	Resolution/scale (coordinate reference system)	Source
Climatic variables						
Mean annual temperature (°C)	5.9	9.2	11.2	Raster	583 m × 927 m (ETRS89/UTM zone 32N) 30 arc second (WGS 84)^a^	Karger et al. (2017, 2018)
Temperature variability (°C)	553.3	624.2	707.4			
Annual precipitation (mm)	443.6	746.8	1765.1			
Precipitation variability (mm)	7.7	17.2	37.4			
Variables from digital elevation model						
Altitude (m a.s.l.)	0	222.2	1080.8	Raster	200 m × 200 m (ETRS89/UTM zone 32N)^a^	GeoBasis‐DE/BKG (2020)
Heat load index	0.68	0.84	0.90			
Land cover classes						
Urban area (%)	0	10.9	100	Raster	100 m × 100 m (ETRS89/UTM zone 32N) 100 m × 100 m (ETRS89/LAEA1052)^a^	EEA/Copernicus programme (2020)
Agricultural area (%)	0	36.7	100			
Area of semi‐natural habitats (%)	0	23.0	99.6			
Forest area (%)	0	25.3	100			
Area of rivers and lakes (%)	0	2.1	100			
Soil variables						
Number of different soil types	1	2.9	8	Vector	1:250,000 (ETRS89/UTM zone 32N)^a^	BGR (2018)

*Note*: Minimum, mean, and maximum values refer to values calculated within the buffer area around each relevé.

^a^ Resolution and coordinate reference system of the original data.

### 2.4 Calculation of functional rarity

As species rarity is scale‐dependent, we quantified FR components for all 218 plant species at three spatial scales using functions from the *funrar* package (Grenié et al., 2017). The local scale refers to the level of each relevé. The regional scale is represented by grid cells of 20 km × 20 km. The national scale is defined as the border of Germany. The size of the grid cells was chosen to guarantee at least two relevés in most of the grid cells (number of relevés per grid cell is between 1 and 239, mean = 15.97, at least two relevés in 85% of the grid cells). Table S6 gives a summary of the measures of FR and its components on the three spatial scales.

Species rarity was measured as scarcity (local scale) and restrictedness (regional and national scale; Grenié et al., 2017; Violle, Thuiller, Mouquet, Munoz, Kraft, Cadotte, Livingstone and Mouillot, 2017). On the local scale, scarcity was quantified for all 218 species in each of the 6500 selected relevés via

(2) $$S_{i}=\exp\left(-N\times A_{i}\times \ln\left(2\right)\right),$$

with *N* being the number of species and *A*_*i*_ the relative abundance of species *i* in a relevé. Scarcity is close to 0 for dominant species and close to 1 for species with very small local population size. If the relative abundance of a species equals 1/*N* (e.g., if all species are equally abundant), scarcity equals 0.5 to prevent species from being classified as either scarce or abundant.

On the regional scale, restrictedness was quantified within grid cells via

(3) $$R_{i}=1-\frac{K_{i}}{K_{\mathrm{tot}}},$$

where *K*_*i*_ is the number of relevés within a grid cell where species *i* occurs and *K*_tot_ is the total number of relevés within this grid cell. Restrictedness equals 0 when species *i* occurs in all relevés within a grid cell and is close to 1 when the species occurs in only few relevés in a grid cell.

On the national scale, restrictedness was quantified as the area of occupancy using Equation (3). Here, *K*_*i*_ represents the number of grid cells where a species occurs and *K*_tot_ the total number of grid cells. Restrictedness equals 0 for widespread species occurring in every grid cell and is close to 1 if a species only occurs in few grid cells.

Functional dissimilarity between all species was calculated via Gower's distance (Gower, 1971), as it can handle different types of variables (here quantitative and categorical traits) and can deal with missing data. We quantified species trait distinctiveness (Grenié et al., 2017; Violle, Thuiller, Mouquet, Munoz, Kraft, Cadotte, Livingstone & Mouillot, 2017) as the mean pairwise functional distance via

(4) $$D_{i}=\frac{\sum_{j=1,j\neq i}^{N}d_{\mathrm{ij}}}{N-1},$$

*d*_*ij*_ being the functional distance between species *i* and *j* scaled by the maximum functional distance in the considered species pool and *N* the number of all species in the considered species pool. Trait distinctiveness equals 0 when all co‐occurring species are functionally identical, i.e., having the same trait values and 1 when species *i* is dissimilar in all traits to all other species *j*, while all species *j* are functionally identical. Trait distinctiveness was calculated for all 218 species on each scale using Equation (4). On the local scale, trait distinctiveness was measured based on all species co‐occurring within a relevé. On the regional scale, all species within a grid cell were used to quantify trait distinctiveness as if they were present in the same community. On the national scale, the whole species set were used to calculate trait distinctiveness.

To identify the functionally rarest species, we calculated the species mean for every FR component on each spatial scale (e.g., mean trait distinctiveness of *A. elatius* across all relevés). We then computed a synthetic index of FR on each scale via

(5) $${\mathrm{FR}}_{i}=\frac{{D_{i}}^{\prime }+{S_{i}}^{\prime }}{2},$$

for the local scale or via

(6) $${\mathrm{FR}}_{i}=\frac{{D_{i}}^{\prime }+{R_{i}}^{\prime }}{2},$$

for the regional and national scale, where *D*_*i*_′ is the mean trait distinctiveness of species *i* scaled between 0 and 1, *S*_*i*_′ is the mean scarcity of species *i* scaled between 0 and 1, and *R*_*i*_′ the mean restrictedness of species *i* scaled between 0 and 1 (note that on the national scale no mean values were calculated as only one value per species and FR component was present). Scaling the mean values of FR components ensures that rarity and trait distinctiveness equally contribute to FR_*i*_. Species with FR_*i*_ close to 1 are among the rarest species that additionally show a very high trait distinctiveness. For an overview of FR_*i*_ and its components per species and scale see Table S7.

To ensure that no trait alone strongly affected the results, we carried out a sensitivity analysis following the procedure by Grenié et al. (2018). We omitted each trait once from the analysis and recalculated *D*_*i*_′ based on the remaining 14 traits as described above for every species at each spatial scale. The results from the reduced trait set were strongly positively correlated with the original results (*r*_S_ > .87, *p* < .001; Figure S1), showing that none of the traits alone heavily affected the results.

### 2.5 Statistical analyses

To determine how species rarity and trait distinctiveness are associated with each other on different spatial scales we calculated the Spearman's *r* (*r*_S_). To analyze which traits drive trait distinctiveness, we performed a distance‐based redundancy analysis (db‐RDA; Legendre & Anderson, 1999) including all complete observations from the species‐trait‐matrix (*n* = 174) and following the procedure by Chapman et al. (2018). For each spatial scale we used the scaled mean trait distinctiveness per species (*D*_*i*_′) as response and the original species‐trait matrix as explanatory variables. We used the Gower's distance metric to account for the non‐numerical structure of some traits. The significance of each trait in the db‐RDA models was assessed via ANOVA by terms using 1000 permutations (Oksanen et al., 2020).

To identify environmental drivers of species richness and FR, we first determined species richness as the number of all species included in the analysis per relevé. We then ranked all species according to their local FR_*i*_ and counted the number of species per relevé that are among the highest 10% of the distribution. As the number of functionally rare species is likely to be higher in species‐rich communities, we followed a null model approach as proposed by Grenié et al. (2018) to address the sampling effect. We simulated 1000 random communities using the curveball algorithm (Strona et al., 2014) and counted the number of functionally rare species occurring in each simulated community. We then calculated the standardized effect size (SES) for each relevé via

(7) $${\mathrm{SES}}_{j}=\frac{X_{j}-Y_{j}^{\prime }}{\mathrm{SD}\;\left(Y_{j}\right)},$$

where *X*_*j*_ is the number of functionally rare species observed in the original relevé *j*, *Y*_*j*_′ is the mean number of functionally rare species from all simulated relevés *j* and SD (*Y*_*j*_) is the standard deviation of the number of functionally rare species from all simulated relevés *j* (Grenié et al., 2018). Values close to 0 show that the occurrence of functionally rare species is mainly driven by species richness, while high negative or positive values indicate that in a community less respectively more functionally rare species occur than expected by chance. The threshold value of 10% was chosen in accordance with Grenié et al. (2018). However, we also checked how results were changed by using threshold values of 5% and 15%. Results were robust as indicated by strong correlation of SES values to the used threshold value of 10% (*r*_S_ between .74 and .81, *p* < .001).

To assess the relationship between species richness and FR, we calculated *r*_S_. We used boosted regression trees (BRTs) to investigate how species richness, the number of functionally rare species, and its SES per relevé depend on 12 environmental variables (Table 2). BRTs and other machine learning approaches represent flexible modeling tools that are increasingly used to explain and predict species distributions (Divíšek & Chytrý, 2018; Thuiller et al., 2006; for a more detailed description see Appendix S2 or Elith et al., 2008). BRT models were fitted using the set of model parameters that minimized predictive deviance (Table S8). Models were simplified by successively dropping the least important predictor variables until predictive deviance of the simplified model exceeded predictive deviance of the original model. To estimate predictive model performance, we used 10‐fold cross‐validation in all BRT models. We performed a permutation test for Moran's *I* statistic to check for spatial autocorrelation in the residuals of the final models within a distance of 38,769 m, so each relevé had at least one neighbor.

All analyses were carried out in R 4.1.2 (R Core Team, 2021) using the following packages: *caret* (Kuhn, 2021), *data.table* (Dowle & Srinivasan, 2021), *dismo* (Hijmans et al., 2021), *doParallel* (Microsoft Corporation & Weston, 2020), *doSNOW* (Microsoft Corporation & Weston, 2022), *fastmatch* (Urbanek, 2021), *funrar* (Grenié et al., 2017), *gbm* (Greenwell et al., 2020), *ggplot2* (Wickham, 2016), *gridExtra* (Auguie, 2017), *profvis* (Chang et al., 2020), *raster* (Hijmans, 2022), *reshape2* (Wickham, 2007), *sf* (Pebesma, 2018), *spdep* (Bivand et al., 2013), *tidyverse* (Wickham et al., 2019), *vegan* (Oksanen et al., 2020), *vegdata* (Jansen & Dengler, 2010).

## 3 RESULTS

### 3.1 Patterns of functional rarity on the species level

**FIGURE 2:**
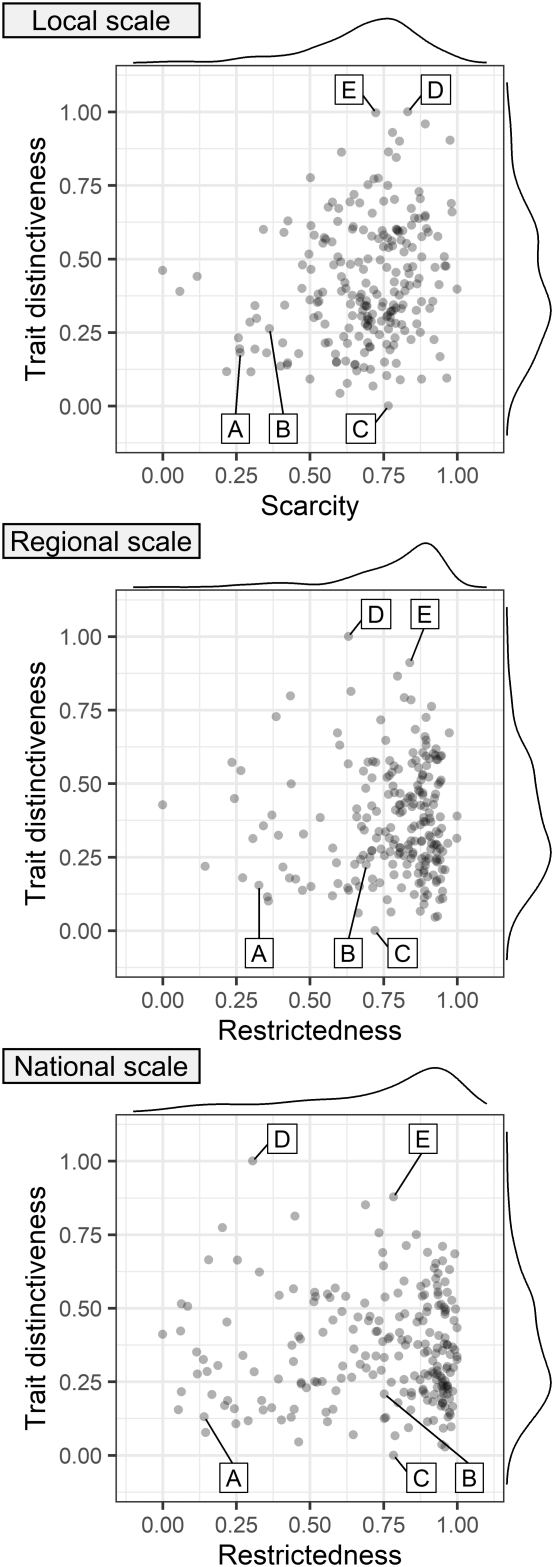
Trait distinctiveness and species rarity in terms of scarcity or restrictedness at different spatial scales show that all combinations are possible apart from high trait distinctiveness and low scarcity at the local scale. Labeled points show the position of five example species: A: *Holcus lanatus*, B: *Salvia pratensis*, C: *Carum carvi*, D: *Vicia cracca*, E: *Vicia hirsuta*. Values for local and regional scale represent species means, calculated from all values at the respective spatial scale. Variables were scaled between 0 and 1. Density plots on the edges represent distribution of the respective variable. Labels of spatial scales were added manually after plotting.

On the local scale, high trait distinctiveness was only observed in rare species as indicated by positive correlation of scarcity and trait distinctiveness (*r*_S_ = .23, *p* < .001; Figure 2). However, on the regional and national scale almost all combinations of species rarity and trait distinctiveness were possible as shown by missing correlation of restrictedness and trait distinctiveness (regional scale: *r*_S_ = .03, *p* > .05; national scale: *r*_S_ = .01, *p* > .05; Figure 2). Across all scales, species rarity (scarcity and restrictedness) was skewed toward high values (Figure 2), i.e., most species were rare and only a few were common, while trait distinctiveness was skewed toward low to medium values (Figure 2), i.e., most species displayed frequent combinations of trait attributes. Figure 2 illustrates the following examples: (i) species that were always rare and had low trait distinctiveness (C — *Carum carvi*), (ii) species that were always rare and had high trait distinctiveness (E — *Vicia hirsuta*), (iii) species that were always common and had low trait distinctiveness (A — *Holcus lanatus*), (iv) species that were locally common but geographically restricted with low trait distinctiveness (B — *Salvia pratensis*), and (v) species that were locally scarce but geographically widespread with distinct trait attributes (D — *Vicia cracca*). Transitions between these classes are smooth.

**FIGURE 3:**
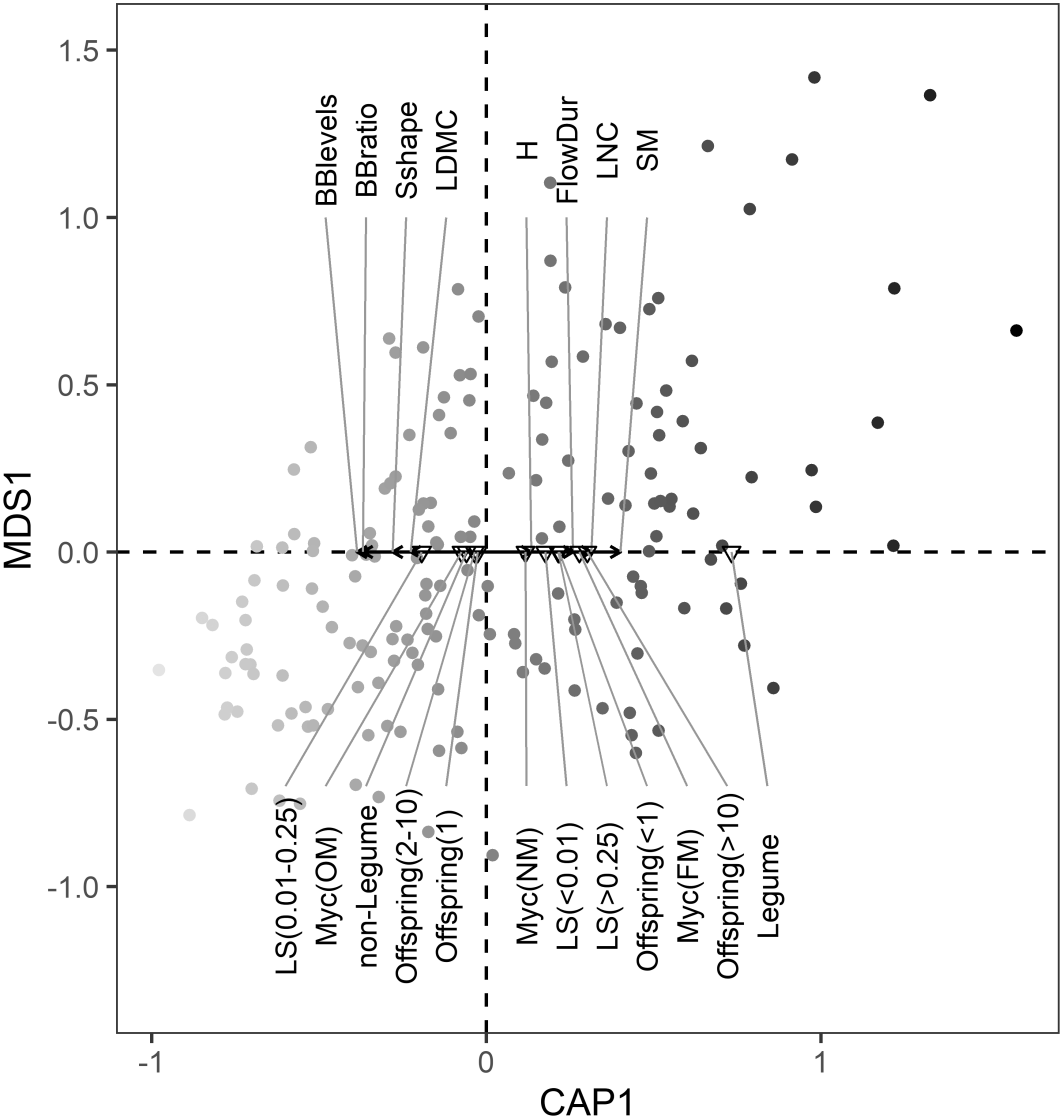
Distance‐based redundancy analysis (db‐RDA) illustrates dissimilarity between species based on their mean scaled local trait distinctiveness and shows traits that drive local trait distinctiveness. Only species with complete trait data for all 15 traits were used in the db‐RDA (*n* = 174). Darker point color represents higher trait distinctiveness. Arrows show the association of numerical traits with the first axis of the db‐RDA while triangles indicate the position of factor levels of categorical traits on the axis. Only significant traits are displayed (ANOVA by terms with 1000 permutations, *p* < .05). BBlevels, number of bud bank levels; BBratio, ratio between number of aboveground vs. belowground bud bank levels; FlowDur, flower duration; H, plant height; LDMC, leaf dry matter content; LNC, leaf nitrogen content per area; LS, maximum lateral spread (horizontal distance: <0.01 m, 0.01–0.25 m, >0.25 m); Myc, mycorrhizal status (FM, facultative mycorrhizal; NM, non‐mycorrhizal; OM, obligate mycorrhizal); Offspring, maximum clonal multiplication rate (number of offspring shoots per parental plant: <1, 1, 2–10, >10); SM, seed mass; Sshape, seed shape.

Figure 3 shows how functional traits were associated with trait distinctiveness on the local scale. Species with low trait distinctiveness were characterized by disk‐like seeds, high leaf dry matter content, obligate mycorrhizal status, a pronounced vegetative regeneration (e.g., higher number of bud bank levels), and intermediate levels of clonality (maximum lateral spread, maximum clonal multiplication rate, and number of clonal growth organs). High trait distinctiveness was associated with legumes, non‐ or facultative mycorrhizal species, tall species with heavy, round seeds, high leaf nitrogen content, long flower duration, and a less pronounced vegetative regeneration. Both, extremely low and high levels of clonality led to higher trait distinctiveness as reflected by clonal multiplication (less than one or more than 10 offspring shoots per parental plant) and lateral spread (<1 cm or more than 25 cm distance lateral spread; Figure 3). Specific leaf area, rooting depth, and the number of clonal growth organs were not associated with trait distinctiveness (ANOVA with 1000 permutations: *p* > .05). On the regional and national scale, patterns for most traits were similar (Figures S2 and S3) except for plant height, which was not associated with trait distinctiveness (ANOVA with 1000 permutations: *p* > .05). In general, trait distinctiveness was highly correlated across spatial scales (*r*_S_ between .97 and .99, *p* < .001).

### 3.2 Patterns of functional rarity and species richness on the community level

Species richness and the number of functionally rare species were positively correlated (*r*_S_ = .28, *p* < .001), i.e., more functionally rare species occurred in species‐rich than in species‐poor communities. On the other hand, species richness and SES of FR were negatively correlated (*r*_S_ = −.30, *p* < .001), i.e., species‐poor communities harbored more functionally rare species than expected by chance and vice versa. BRT models explained 52%–75% of variance in species richness and FR using environmental variables (species richness: cross‐validation correlation mean = 0.750; number of functionally rare species: cross‐validation correlation mean = 0.516; SES of FR: cross‐validation correlation mean = 0.571). Residuals of the models showed no spatial autocorrelation (Moran's *I* between −0.009 and −0.006, *p* > .05).

**FIGURE 4:**
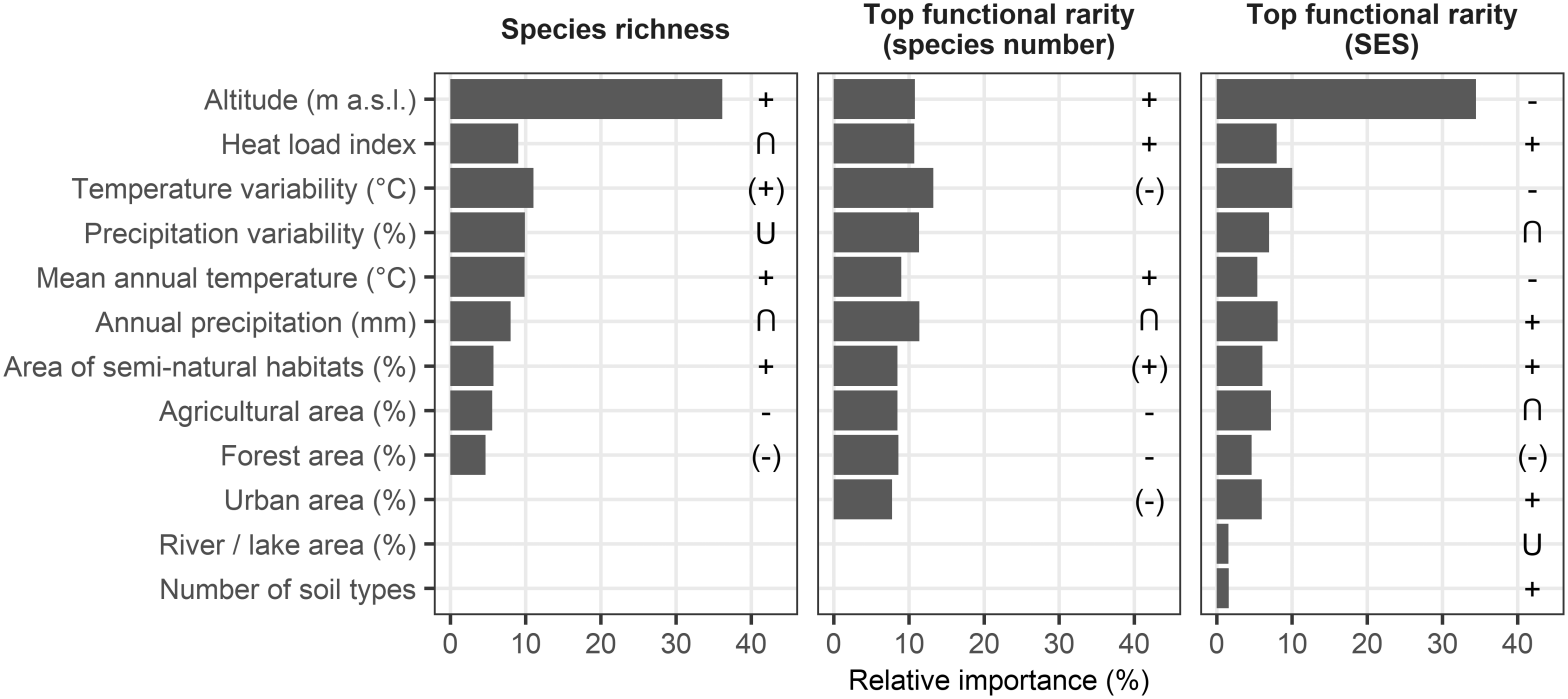
Relative importance of environmental variables in boosted regression tree (BRT) models for species richness and functional rarity of all species in the analysis. Symbols represent the observed shape of the relationship between response (given on top of the subplots) and explanatory variables derived from partial dependency plots (+ positive, − negative, U‐shaped, ∩ hump‐shaped relationship; Figures S4–S6). Positive and negative relationships are mostly non‐linear with brackets indicating a less clear relationship. Missing symbols illustrate seemingly arbitrary relationships. Hump symbols were added manually after plotting.

For species richness, altitude was by far the most important explanatory variable, while all other variables were less and approximately equally important (Figure 4). Species richness strongly increased with altitude, reaching a plateau at 300 m (Figure S4). For temperature and precipitation variability relationships were slightly positive or U‐shaped, respectively. Species richness decreased for low and high levels of heat load whereas it increased with mean annual temperature, and showed a hump‐shaped relationship with annual precipitation having its maximum around 500–600 mm per year (Figure 4, Figure S4). While a higher proportion of semi‐natural habitats in the surrounding increased species richness, more agricultural or forest areas led to lower species richness (Figure 4).

For the number of functionally rare species, all environmental variables were similarly important in the BRT model (Figure 4). The number of functionally rare species increased with altitude and heat load but decreased with temperature variability (Figure 4). For precipitation variability no clear patterns were observed (Figure 4, Figure S5). Mean annual temperature and annual precipitation relationships were positive or hump‐shaped, respectively. The number of functionally rare species slightly increased with the proportion of semi‐natural habitats in the surrounding and decreased with a higher proportion of agricultural, forest, and urban area (Figure 4).

For the SES of FR, altitude was the most important predictor variable while all others were less important (Figure 4). More functionally rare species than expected by chance occurred in areas of low altitude or areas characterized by higher heat load, lower temperature variability and intermediate precipitation variability (Figure 4, Figure S6). More functionally rare species than expected by chance were also found in colder or wetter habitats or in relevés surrounded by a large proportion of agricultural areas (Figure 4, Figure S6). SES of FR was higher for relevés surrounded by a large proportion of semi‐natural habitats and lower for relevés surrounded by a large proportion of forest areas. With increasing proportion of urban area or increasing number of soil types in the surrounding, the number of functionally rare species was higher than expected by chance (Figure 4).

## 4 DISCUSSION

For the first time, we investigated patterns and drivers of plant FR in German hay meadows to better understand FR on the species and community level. In the following, we argue that most functionally rare species were transients from surrounding habitats. Although these species are not rare per se, they were rare in the selected habitat due to the display of functionally distinct but maladaptive trait attributes. This is also reflected by the preference of functionally rare species for environmental conditions that strongly differed from conditions resulting in species‐rich hay meadows. In consequence, we observed more functionally rare species than expected by chance in species‐poor communities. Further, we argue that more functionally rare species occurred if empty niche space was available and if suitable environmental conditions and source habitats in the surrounding were present.

### 4.1 Patterns of functional rarity on the species level

On the local scale high trait distinctiveness was only observed in rare species while on the regional and national scale both rare and common species showed high trait distinctiveness. Low trait distinctiveness was observed in rare and common species on all spatial scales. Species with frequent trait attributes, i.e., traits that are typically observed in grassland species (e.g., pronounced vegetative regeneration or clonality), showed low trait distinctiveness while infrequent trait attributes led to high trait distinctiveness. We argue that these patterns are in line with ecological theory (environmental filtering, niche differentiation) and that the relationship between rarity and trait distinctiveness is closely linked to the traits that drive trait distinctiveness of species in hay meadows (e.g., a less pronounced vegetative regeneration can lead to high trait distinctiveness, but may be disadvantageous in frequently mown hay meadows, resulting in higher rarity).

Environmental filtering predicts that abiotic conditions select for an optimal trait set, which is beneficial in the current environment (Maire et al., 2012; Umaña et al., 2015). This optimal trait set should be located at the center of the trait space (Umaña et al., 2015), represented by low trait distinctiveness. Indeed, most of the trait attributes that led to low trait distinctiveness reflect strategies that are advantageous in frequently disturbed grasslands. For example, a short flower duration allows for fast sexual reproduction between mowing events in highly disturbed habitats (Grime, 1974); vegetative bud banks and clonal growth allow for fast regeneration (Klimešová et al., 2018; Klimešová & Klimeš, 2007) and space occupancy after disturbance (Herben et al., 2018; Klimešová et al., 2018, 2019); and obligate mycorrhizal species may have a competitive advantage over non‐mycorrhizal plants as they receive additional nutrients from their symbiotic partner (van der Heijden et al., 2015). We found some species with this optimal trait set to be dominant (i.e., low scarcity and low trait distinctiveness), supporting the environmental filtering theory. On the other hand, limiting similarity proposes that more similar species suffer from higher competition leading to lower abundance (MacArthur & Levins, 1967; Mouillot et al., 2007). The presence of many species with low trait distinctiveness and high scarcity shows that both processes may act simultaneously.

To avoid competition, species may diverge from the optimal trait set (Maire et al., 2012), leading to infrequent trait attributes and consequently higher trait distinctiveness. We think that locally rare species with high trait distinctiveness represent either specialist or transient species (Chapman et al., 2018; Umaña et al., 2015) — a distinction that is also suited to explain why rarity and trait distinctiveness are uncorrelated on larger spatial scales. To avoid competitive exclusion, specialists may focus on using a particular but rare resource which may also limit local abundance (Chapman et al., 2018; Gaston, 1994) leading to high scarcity. Still, occupying a niche that is available throughout the habitat facilitates a constant occurrence within the habitat, leading to low restrictedness (e.g., *Vicia cracca*). Transient species originate from surrounding habitats (Grime, 1998) and should display a trait set that is tuned to fit environmental conditions of the source habitat. This trait set may be maladaptive in hay meadows, leading to lower performance and consequently to high local rarity (Umaña et al., 2015). We argue that transient species lack a permanent niche in the sink habitat and consequently occur only occasionally, leading to high restrictedness at higher spatial scales (e.g., *Galium aparine*). However, transient species are not geographically restricted per se but only when focusing on a certain habitat type (e.g., *Galium aparine* occurs throughout Germany; Bettinger et al., 2013). In addition, this explanation does not hold for all species as there are also non‐transients that are rare at all spatial scales (e.g., *Colchicum autumnale*). This indicates the importance of other processes on larger spatial scales for species distribution (e.g., availability in the regional species pool linked to dispersal; Götzenberger et al., 2012).

Although some studies already investigated the relationship between rarity and trait distinctiveness across different organism groups (Chapman et al., 2018; Grenié et al., 2017; Mouillot et al., 2013; Umaña et al., 2015), most of them are limited to one spatial scale (but see Mouillot et al., 2013) or do not explain the occurrence of the observed relationship (but see Chapman et al., 2018; Umaña et al., 2015). Our findings stress the potential of FR to better understand habitat‐specific assembly processes and species distributions, especially if more than one spatial scale is considered and functional traits are used to explain the occurring patterns.

### 4.2 Patterns of functional rarity and species richness on the community level

More functionally rare species occurred in species‐rich than in species‐poor communities. Yet, species‐poor communities hosted more functionally rare species than expected by chance. In the following, we first provide a general explanatory approach about how the sampling effect and higher niche overlap in species‐rich communities may cause these patterns. Using environmental variables, we then show that functionally rare species occur under environmental conditions that differ from conditions typical of species‐rich hay meadows.

The relationships between environmental variables and the observed number of functionally rare species were largely consistent with the relationships between environmental variables and species richness. In consequence, species‐rich relevés contained more functionally rare species than species‐poor assemblages. We attribute this finding to the sampling effect (Huston, 1997; Tilman et al., 1997), i.e., more diverse communities have a higher probability of functionally rare species occurring there. When accounting for the sampling effect, species‐poor communities hosted more functionally rare species than expected by chance and vice versa. This is in line with Grenié et al. (2018) and Trindade‐Santos et al. (2022), who observed the same mismatch between species richness and the SES of FR for fish species on a global scale. Yet, explanations cannot be easily transferred to our findings as we focus on a different organism group, a particular habitat type and a smaller spatial scale where different process may act (Götzenberger et al., 2012). We speculate that in locally species‐rich communities establishment success of functionally rare species is reduced as the niche space is more filled. This is particularly important as many functionally rare species are probably transients that originate from the surrounding vegetation. In species‐poor communities more niches are likely unoccupied, facilitating colonization by functionally rare species. This speculation is supported by Mwangi et al. (2007), who transplanted native grassland species into grassland communities of different species richness levels and showed that the biomass of transplanted species decreased under higher species richness. They attribute this pattern to higher niche overlap, i.e., stronger competition, between transplanted species and the target community caused by a more filled niche space in species‐rich communities. However, this explanation contradicts the definition of FR; as functionally rare species display distinct trait attributes, niche overlap between functionally rare species and the target community should be rather low.

We identified topographic and climate variables as main drivers of species richness in hay meadows, which is in line with previous modeling approaches (Divíšek & Chytrý, 2018; Irl et al., 2015). Species richness decreased for lowland areas below 300 m, which predominantly occur in the northern and northeastern parts of Germany. This region is among the most intensively managed areas of Europe (Olesen & Bindi, 2002), with grasslands predominantly used for biomass production in livestock and dairy farming (BMEL, 2020). Intensive land use, i.e., high cutting frequency and fertilization, leads to reduced species richness in semi‐natural grasslands (Kleijn et al., 2009; Socher et al., 2012). In addition, higher ground water tables in this region lead to the formation of wetter hay meadow associations, which are less speciose (Leuschner & Ellenberg, 2018). Surprisingly, species richness increased with higher climatic variability. In general, it is assumed that only few species survive unpredictable climatic conditions (environmental stability hypothesis; Klopfer, 1959). However, stronger climatic variation may not occur randomly but in terms of predictable seasons. This way variability could promote the coexistence of species by providing different ecological niches over time (Pausas & Austin, 2001). The hump‐shaped relationship of species richness to heat load and annual precipitation indicates optimal growing conditions for most species at intermediate water and energy availability (Pausas & Austin, 2001). While under low annual precipitation or high heat load limited water availability may negatively affect plant performance, only few species that are adapted to wet site conditions may survive under high annual precipitation. Under low heat load, e.g., on steeper Northwest exposed sites, low energy availability may limit plant growth. If water is not limiting, species richness is expected to increase with annual temperature (Pausas & Austin, 2001), as shown here. Species richness increased with higher connectivity and reduced dispersal limitations between grasslands (Divíšek & Chytrý, 2018) as indicated by larger proportion of semi‐natural and smaller proportion of forest and agricultural area in the surrounding.

The relationships between environmental variables and the SES of FR contradicted relationships between environmental variables and species richness. In consequence, environmental conditions that led to low species richness supported the occurrence of more functionally rare species than expected by chance. This is due to species‐specific habitat preferences of functionally rare species, many of which were transient species from other habitats. For example, the proposed high land use intensity at low altitudes may lead to low species richness. However, disturbance via mowing creates gaps in the vegetation structure and reduces competition (Grime, 2006). These gaps could be used by functionally rare species from the surrounding landscape to colonize the habitat. More functionally rare species than expected by chance occurred under low temperature and high precipitation indicating a preference of some functionally rare species for wet growing conditions (e.g., *Equisetum palustre*, *Persicaria amphibia*; Müller et al., 2021). This is also partly supported by unexpectedly high numbers of functionally rare species observed for low altitudes, where wetter hay meadow associations occur (Leuschner & Ellenberg, 2018). In contrast to species richness, both the observed and expected number of functionally rare species increased with heat load and decreased with temperature variability. This indicates a preference of functionally rare species for climatically stable conditions but higher drought stress.

In addition to habitat preferences, relevés with a higher proportion of source habitats in the surrounding hosted more functionally rare species than expected by chance. Many functionally rare species were arable weeds or species of often disturbed, ruderal areas (e.g., *Stellaria media*, *Galium aparine*, *Capsella bursa‐pastoris*, *Allium vineale*, *Urtica dioica*, *Silene latifolia*; Appendix S3 of Chytrý et al., 2020). In consequence, more functionally rare species than expected by chance occurred in relevés with a higher proportion of agriculturally used or urban area in the surrounding. In accordance with patterns of species richness, both the observed number of functionally rare species and the SES of FR increased with semi‐natural areas and decreased with forest areas in the surrounding. We attribute the consistent positive effect of semi‐natural areas to the close association of some species with frequently managed grassland habitats (e.g., *Bellis perennis*, *Vicia cracca*; Appendix S3 of Chytrý et al., 2020). The negative effect of forest areas likely results from the low number of forest species in the list of functionally rare species.

### 4.3 Limitations

The findings of our study should be viewed with respect to some limitations. First, we expect the distribution of trait values in a species set to affect the trait distinctiveness of the individual species. Original trait values are often skewed, which leads to an aggregation of species in a certain region of the multidimensional trait space. A broad and uniform distribution of trait values may lead to a more balanced calculation of trait distinctiveness as distances between species are evenly spaced. For continuous traits, this distribution could be achieved via ranking species by their trait values. Though this transformation keeps the order of the species along a trait axis, information on the distance between measured trait values are lost. Transforming trait values to follow a more normal distribution, e.g., by log‐transformation, maintains the trait‐specific distances between species, but on a different scale. In consequence, the impact of outlier values for trait distinctiveness calculation would be reduced. However, functionally rare species are defined as “ecological outliers” (Violle, Thuiller, Mouquet, Munoz, Kraft, Cadotte, Livingstone & Mouillot, 2017). As we assume that environmental filters rather act on the expression of original trait values than on transformed values, we used untransformed trait values in our analysis. However, future studies should investigate how the transformation of trait values affects the calculation of FR and associated ecological findings.

Second, we excluded all species from the analyses that occurred in <1% of the selected relevés assuming that these do not represent rare hay meadow species sensu stricto (see methods section). We selected this threshold as an unsupervised method to exclude species that were likely randomly associated with the selected habitat type (Bruelheide, 2016). Though such threshold values provide valuable benefits (e.g., higher robustness of the results in case of species misidentification; less susceptibility of scarcity calculation to outliers in abundance estimation), they should be used with caution. In our case, indeed, the application of the threshold reliably excluded the majority of species being not characteristic of hay meadows (based on Appendix S3 of Chytrý et al., 2020). Still, we argued that many of the species included in the analyses, and especially most of the functionally rare ones, were transients. In addition, the application of the threshold value excluded species from the analysis that are rare in terms of occurrence. However, assuming that the omitted species were randomly occurring transients, we do not expect our conclusions to change. Including all species would then only add more transients to the dataset, which might even reinforce the observed patterns, especially on the regional and national scale.

With an explained variance of more than 50%, our BRT models were well‐suited to model patterns of species richness and FR depending on environmental variables. However, additional variables (e.g., relevé size, microclimate, land use) may increase the predictive power and facilitate an even better understanding of the observed spatial patterns. Unfortunately, information on relevé size were not consistently available for the analyses. Yet, relevé size is known to affect species richness (Divíšek & Chytrý, 2018), which in turn could also affect the occurrence of rare species and thus FR. For our analysis, we assumed that vegetation ecologists chose an appropriate relevé size at which species richness saturates. In addition, a comparison of our original species richness model and a species richness model for a subset where relevé size was available showed similar relationships between richness and environmental variables across the models (Appendix S4). However, relevé size may still influence species richness and the observed number of functionally rare species (Appendix S4). Here, we additionally accounted for this effect by calculating the SES for the number of functionally rare species.

Finally, we argued that areas of low altitude are more intensively used than high‐elevation areas. Of course, local information on land use would yield less speculative results. However, consistent data on current or historical land use are hardly available for small spatial scales, not to mention national levels.

## 5 CONCLUSION

Grenié et al. (2018) and Trindade‐Santos et al. (2022) showed for fish species on a global scale that species‐poor areas host more functionally rare species than expected by chance indicating that we do not protect all facets of biodiversity. In consequence, Grenié et al. (2018) suggested using FR as an additional prioritization criterion for nature conservation. Although we confirmed this mismatch on a local scale for plant species in hay meadows, our findings do not support the relevance of this mismatch for nature conservation. In our study, functionally rare species were mostly transient species that are not endangered or rare on larger spatial scales. Studies of FR that focus on the biogeographical distribution of plant species of a certain habitat type, may come to similar conclusions as Grenié et al. (2018) or Trindade‐Santos et al. (2022). However, we argue that species rarity and trait distinctiveness are highly habitat specific. In our case, the focus on the local scale revealed that apart from nature conservation FR can also be a useful tool to better understand why species are rare and under which conditions these species occur.

## AUTHOR CONTRIBUTIONS

**Gabriel Walther:** Conceptualization (equal); formal analysis (lead); investigation (lead); methodology (lead); visualization (lead); writing – original draft (lead); writing – review and editing (equal). **Ute Jandt:** Methodology (supporting); writing – review and editing (equal). **Jens Kattge:** Writing – review and editing (equal). **Christine Römermann:** Conceptualization (equal); methodology (supporting); writing – review and editing (equal).

## CONFLICT OF INTEREST

The authors declare no conflicts of interest.

## Supporting information


Figures S1–S6

Tables S1–S9

Appendix S1

Appendix S2

Appendix S3

Appendix S4

## Data Availability

Vegetation data from the German Vegetation Reference Database, trait data from BiolFlor and gap‐filled trait data from TRY are not publicly available due to privacy or ethical restrictions. Trait data from CLO‐PLA, GRooT, observed trait data from TRY, climate data from CHELSA, Corine land cover data as well as the digital elevation model and the soil map of Germany are publicly available. A detailed description of how to access all used data can be found on Dryad together with the R code used in the analysis and selected derived data: https://doi.org/10.5061/dryad.zpc866tc1.
