# Supplementary material for: Functional rarity of plants in German hay meadows — Patterns on the species level and mismatches with community species richness
Source: Ecol Evol. 2022 Oct 1;12(10):e9375. doi: 10.1002/ece3.9375 (PMC9526122; doi:10.1002/ece3.9375)
Supplement: Supplementary file 1 — Figures S1–S6 [file ECE3-12-e9375-s001.docx]

# Supplementary figures


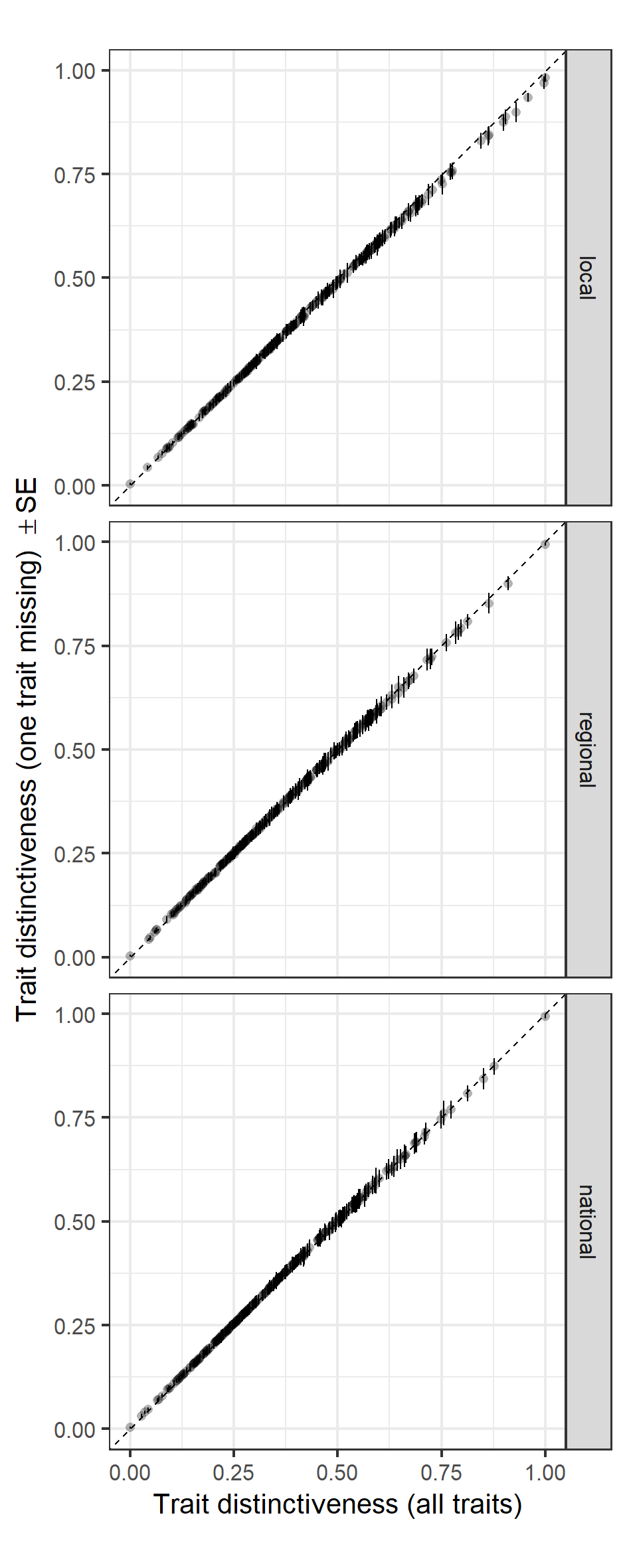


Fig. S1 Results from the sensitivity analysis comparing mean scaled trait distinctiveness per species for all 15 selected traits and after omitting each trait once and recalculating mean scaled trait distinctiveness per species and spatial scale. Points represent mean values of recalculated trait distinctiveness and error bars represent standard error of recalculated trait distinctiveness.


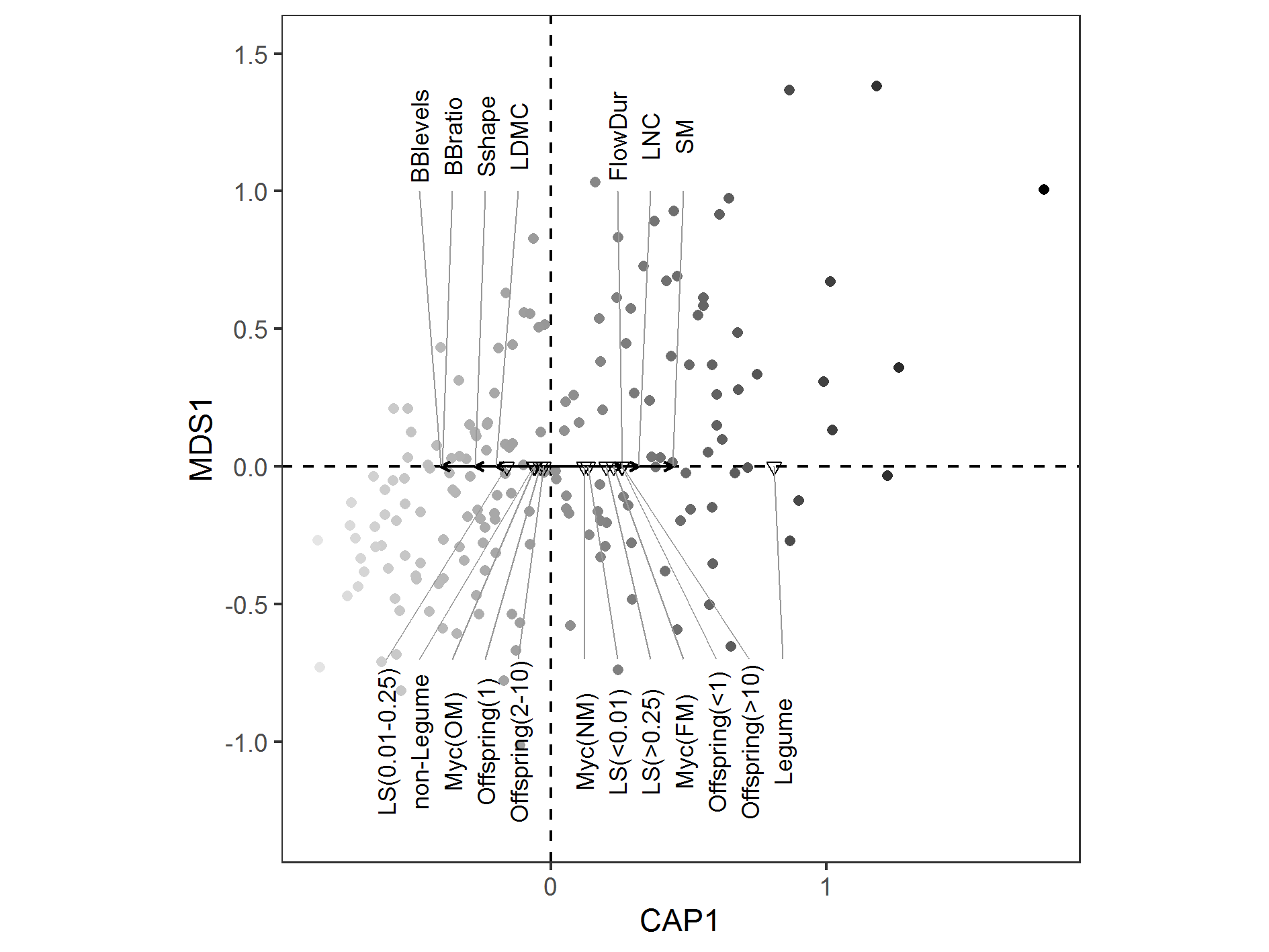


Fig. S2 Distance-based redundancy analysis (db-RDA) illustrates dissimilarity between species based on their mean scaled regional trait distinctiveness and shows traits that drive regional trait distinctiveness. Only species with complete trait data for all 15 traits were used in the db-RDA (n = 174). Darker point color represents higher trait distinctiveness. Arrows show the association of numerical traits with the first axis of the db-RDA while triangles indicate the position of factor levels of categorical traits on the axis. Only significant traits are displayed (ANOVA by terms with 1000 permutations, p < 0.05). Abbreviations: LDMC – leaf dry matter content, LNC – leaf nitrogen content per area, SM – seed mass, Sshape – seed shape, FlowDur – flower duration, LS – maximum lateral spread maximum lateral spread (horizontal distance: <0.01m, 0.01–0.25m, >0.25m), Offspring – maximum clonal multiplication rate (number of offspring shoots per parental plant: <1, 1, 2-10, >10), BBlevels – number of bud bank levels, BBratio – ratio between number of aboveground vs. belowground bud bank levels, Myc – mycorrhizal status (FM – facultative mycorrhizal, NM – non-mycorrhizal, OM – obligate mycorrhizal).


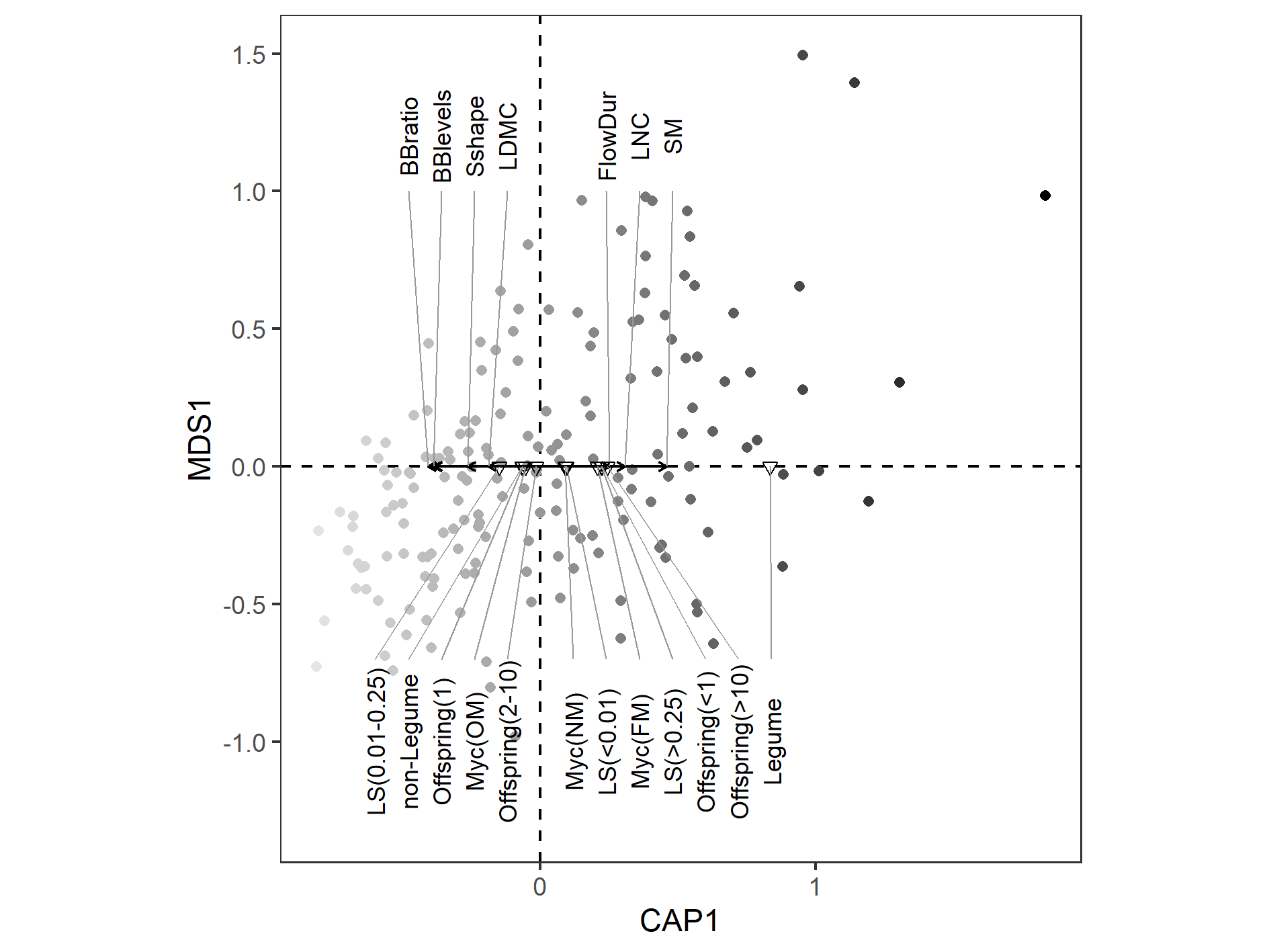


Fig. S3 Distance-based redundancy analysis (db-RDA) illustrates dissimilarity between species based on their scaled national trait distinctiveness and shows traits that drive national trait distinctiveness. Only species with complete trait data for all 15 traits were used in the db-RDA (n = 174). Darker point color represents higher trait distinctiveness. Arrows show the association of numerical traits with the first axis of the db-RDA while triangles indicate the position of factor levels of categorical traits on the axis. Only significant traits are displayed (ANOVA by terms with 1000 permutations, p < 0.05). Abbreviations: LDMC – leaf dry matter content, LNC – leaf nitrogen content per area, SM – seed mass, Sshape – seed shape, FlowDur – flower duration, LS – maximum lateral spread maximum lateral spread (horizontal distance: <0.01m, 0.01–0.25m, >0.25m), Offspring – maximum clonal multiplication rate (number of offspring shoots per parental plant: <1, 1, 2-10, >10), BBlevels – number of bud bank levels, BBratio – ratio between number of aboveground vs. belowground bud bank levels, Myc – mycorrhizal status (FM – facultative mycorrhizal, NM – non-mycorrhizal, OM – obligate mycorrhizal).


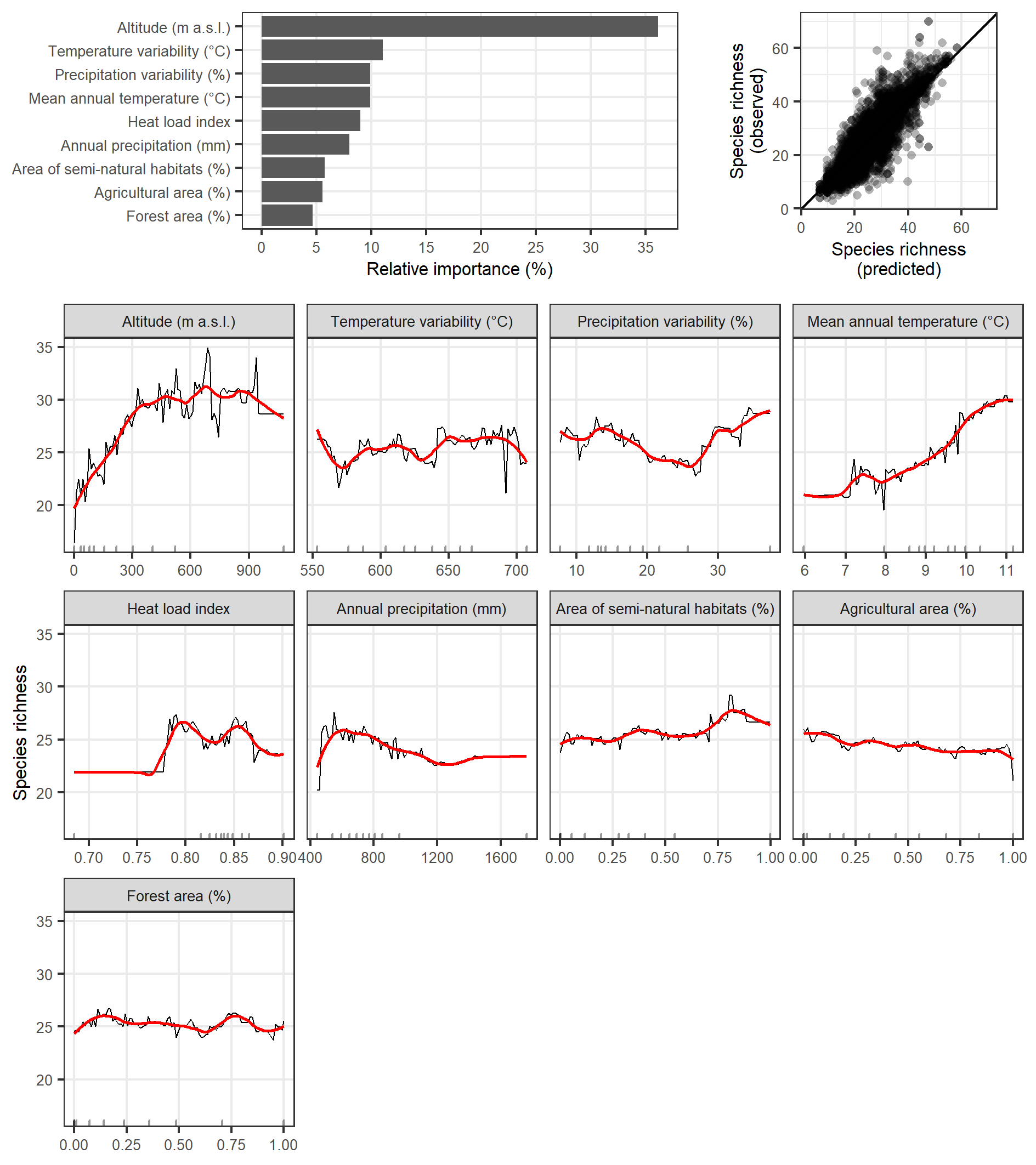


Fig. S4 Results from the final boosted regression tree model for species richness per relevé. Upper plot row shows relative importance of the predictive variables included in the final model (left) and relationship between observed and predicted values from the final model (right). Lower plot rows show partial dependence plots for predictive variables included in the final model, ordered by their relative importance (decreasing from top left to bottom right). Black line: fitted relationship between response and predictive variable; red line: smooth representation of fitted relationship; tick marks on x-axes: 10 percent quantiles for values of the predictive variables.


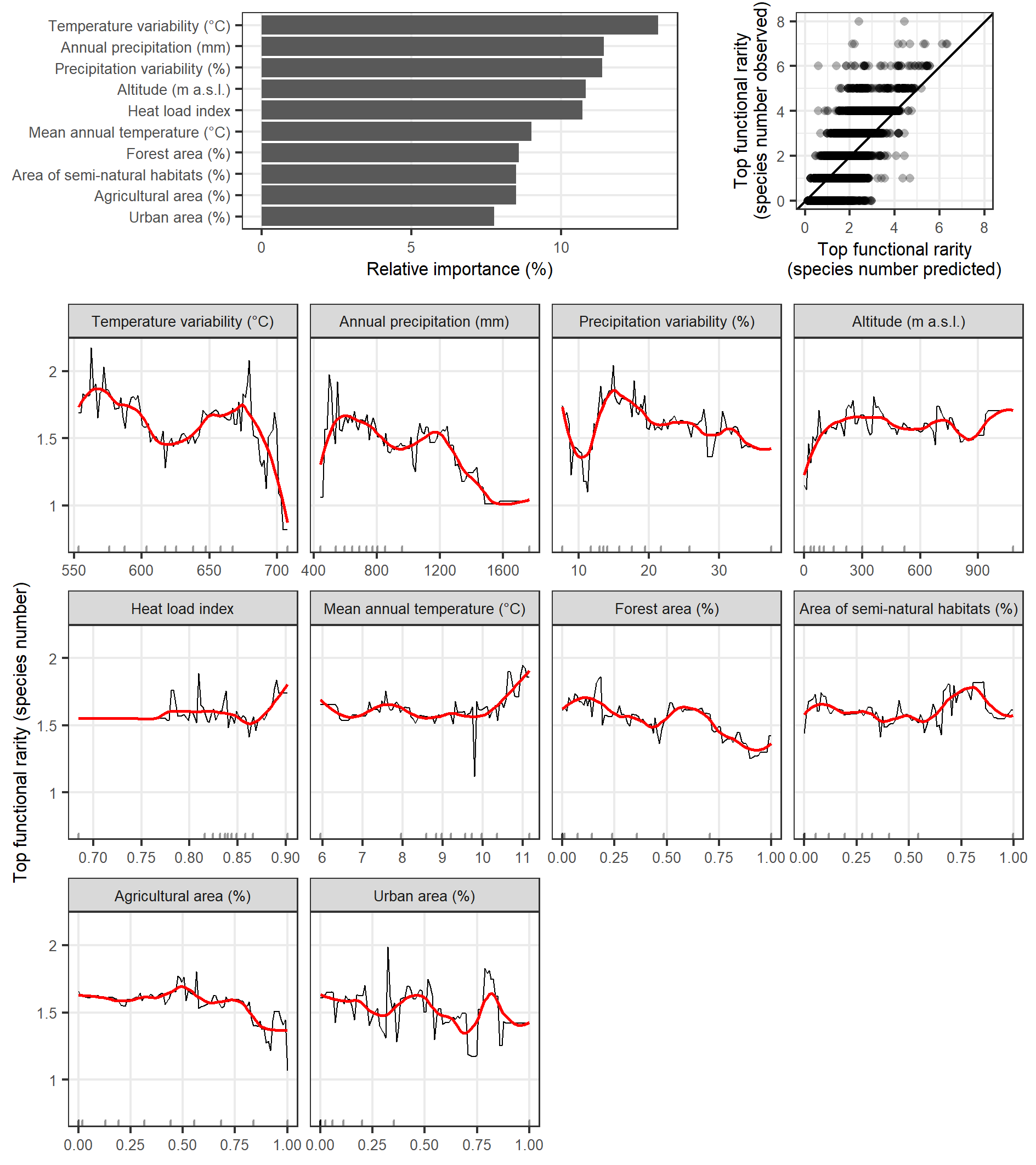


Fig. S5 Results from the final boosted regression tree model for the observed number of functionally rare species per relevé. Upper plot row shows relative importance of the predictive variables included in the final model (left) and relationship between observed and predicted values from the final model (right). Lower plot rows show partial dependence plots for predictive variables included in the final model, ordered by their relative importance (decreasing from top left to bottom right). Black line: fitted relationship between response and predictive variable; red line: smooth representation of fitted relationship; tick marks on x-axes: 10 percent quantiles for values of the predictive variables.


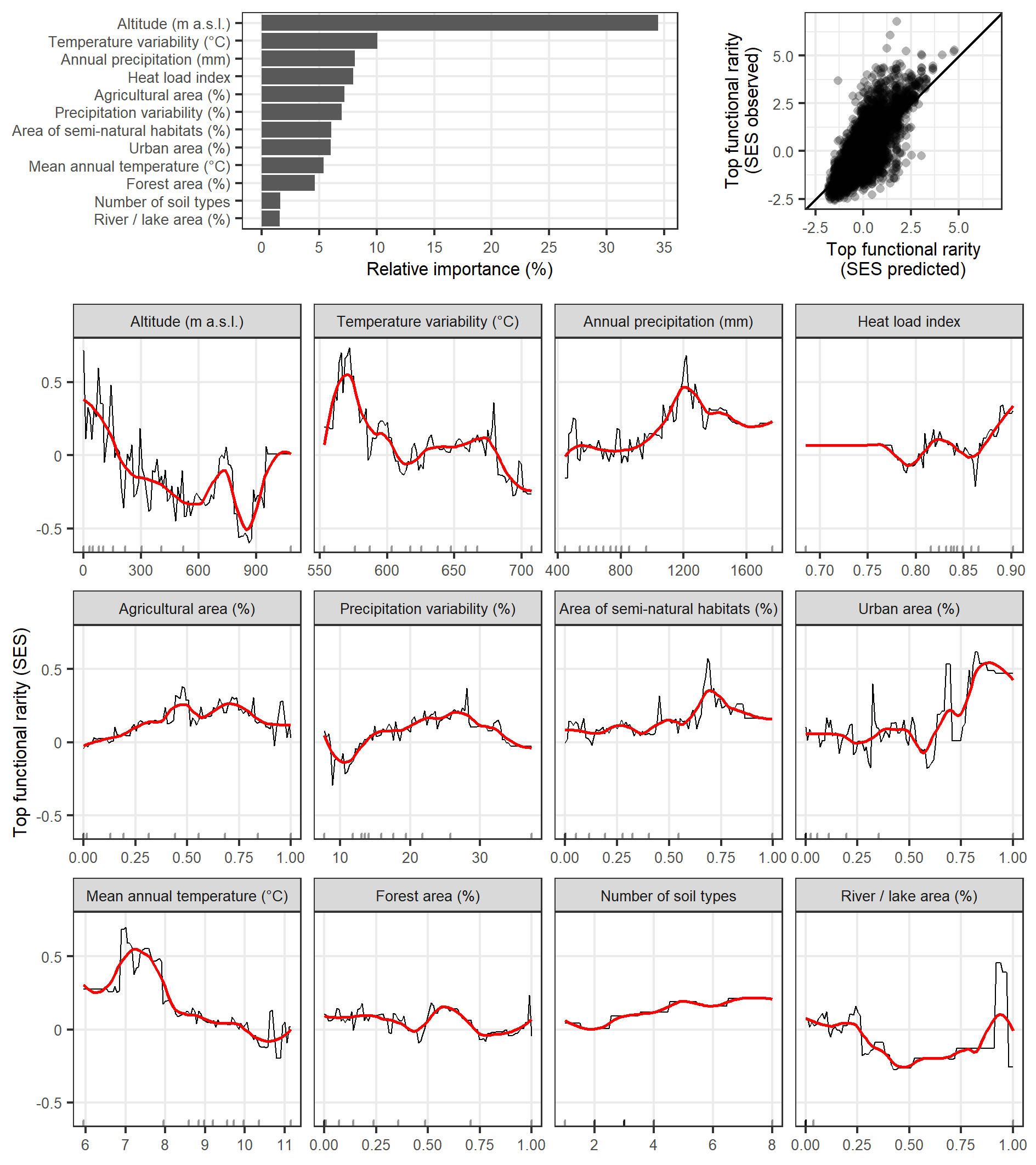


Fig. S6 Results from the final boosted regression tree model for the standardized effect size of the number of functionally rare species per relevé. Upper plot row shows relative importance of the predictive variables included in the final model (left) and relationship between observed and predicted values from the final model (right). Lower plot rows show partial dependence plots for predictive variables included in the final model, ordered by their relative importance (decreasing from top left to bottom right). Black line: fitted relationship between response and predictive variable; red line: smooth representation of fitted relationship; tick marks on x-axes: 10 percent quantiles for values of the predictive variables.
