# Supplementary material for: Functional rarity of plants in German hay meadows — Patterns on the species level and mismatches with community species richness
Source: Ecol Evol. 2022 Oct 1;12(10):e9375. doi: 10.1002/ece3.9375 (PMC9526122; doi:10.1002/ece3.9375)
Supplement: Supplementary file 2 — Tables S1–S9 [file ECE3-12-e9375-s004.zip › ece39375-sup-0002-TableS1-S9.docx]

# Supplementary Tables

Tab. S1 Results of Spearman correlation of traits contained in species-trait matrix. Upper triangle contains Spearman’s r. Lower triangle contains p-values. Values in bold indicate significant relationship (p ≤ 0.05).

|  | Specific leaf area | Leaf dry matter content | Leaf N per area | Plant height | Seed mass | Seed shape | Rooting depth | Flower duration | Maximum lateral spread | Maximum clonal multiplication rate | Number of bud bank levels | Ratio between number of aboveground vs. belowground bud bank levels | Number of clonal growth organs | Legume | Mycorrhizal status |
| --- | --- | --- | --- | --- | --- | --- | --- | --- | --- | --- | --- | --- | --- | --- | --- |
| Specific leaf area |  | **-0.335** | **-0.475** | -0.027 | **-0.267** | **-0.139** | **-0.264** | 0.057 | 0.083 | 0.048 | 0.103 | 0.002 | **0.216** | 0.036 | -0.094 |
| Leaf dry matter content | < 0.001 |  | 0.064 | 0.059 | 0.011 | 0.078 | **0.15** | **-0.319** | -0.076 | **0.189** | 0.029 | **0.159** | -0.031 | **0.136** | **0.165** |
| Leaf N per area | < 0.001 | 0.348 |  | **0.153** | **0.377** | **-0.157** | **0.234** | 0.047 | -0.005 | -0.056 | -0.059 | **0.140** | **-0.143** | **0.366** | 0.131 |
| Plant height | 0.695 | 0.387 | 0.024 |  | **0.232** | 0.119 | **0.318** | **-0.180** | 0.041 | 0.077 | 0.036 | **0.356** | 0.058 | 0.069 | -0.007 |
| Seed mass | < 0.001 | 0.867 | < 0.001 | < 0.001 |  | -0.084 | **0.305** | -0.097 | -0.066 | **-0.148** | -0.121 | -0.011 | 0.049 | **0.227** | 0.136 |
| Seed shape | 0.040 | 0.254 | 0.021 | 0.080 | 0.219 |  | 0.069 | -0.095 | -0.019 | -0.025 | 0.054 | 0.044 | -0.072 | **-0.355** | **0.295** |
| Rooting depth | < 0.001 | 0.026 | < 0.001 | < 0.001 | < 0.001 | 0.310 |  | -0.111 | 0.074 | -0.106 | -0.135 | **0.275** | -0.062 | 0.047 | -0.060 |
| Flower duration | 0.408 | < 0.001 | 0.488 | 0.008 | 0.154 | 0.165 | 0.103 |  | -0.083 | -0.068 | **-0.179** | -0.090 | -0.013 | 0.015 | -0.011 |
| Maximum lateral spread | 0.246 | 0.286 | 0.944 | 0.570 | 0.356 | 0.796 | 0.302 | 0.249 |  | **0.306** | **0.203** | **0.200** | -0.055 | -0.088 | -0.131 |
| Maximum clonal multiplication rate | 0.514 | 0.009 | 0.442 | 0.292 | 0.042 | 0.736 | 0.144 | 0.356 | < 0.001 |  | **0.351** | **0.204** | **-0.176** | 0.066 | **-0.154** |
| Number of bud bank levels | 0.162 | 0.694 | 0.427 | 0.625 | 0.100 | 0.462 | 0.067 | 0.015 | 0.006 | < 0.001 |  | -0.007 | -0.015 | 0.058 | 0.038 |
| Ratio between number of aboveground vs. belowground bud bank levels | 0.981 | 0.020 | 0.040 | < 0.001 | 0.868 | 0.522 | < 0.001 | 0.192 | 0.005 | 0.005 | 0.923 |  | -0.035 | -0.015 | -0.012 |
| Number of clonal growth organs | 0.001 | 0.647 | 0.036 | 0.398 | 0.470 | 0.295 | 0.363 | 0.852 | 0.443 | 0.016 | 0.840 | 0.606 |  | 0.116 | -0.027 |
| Legume | 0.601 | 0.045 | < 0.001 | 0.308 | < 0.001 | < 0.001 | 0.486 | 0.821 | 0.217 | 0.368 | 0.436 | 0.830 | 0.088 |  | **0.140** |
| Mycorrhizal status | 0.181 | 0.019 | 0.063 | 0.917 | 0.053 | < 0.001 | 0.398 | 0.872 | 0.073 | 0.038 | 0.620 | 0.862 | 0.700 | 0.045 |  |

Tab. S2 Species-trait-matrix for 218 selected species from lowland hay meadows and 15 ecologically relevant traits.

Please find the table attached as .xlsx-file “Tab_S2_Walther_et_al.xlsx”

Tab. S3 Data summary for continuous traits.

| **Trait [unit]** | **Min** | **5 %** | **25 %** | **Mean** | **Median** | **75 %** | **95 %** | **Max** | **NA** |
| --- | --- | --- | --- | --- | --- | --- | --- | --- | --- |
| Specific leaf area | 8.975 | 13.879 | 19.045 | 23.582 | 22.485 | 27.085 | 36.998 | 48.587 | 0 |
| Leaf dry matter content | 0.086 | 0.133 | 0.181 | 0.227 | 0.225 | 0.27 | 0.349 | 0.394 | 0 |
| Leaf N per area | 0.519 | 0.687 | 0.946 | 1.234 | 1.186 | 1.424 | 2.018 | 2.829 | 0 |
| Plant height | 0.046 | 0.106 | 0.217 | 0.398 | 0.344 | 0.529 | 0.869 | 1.462 | 0 |
| Seed mass | 0.025 | 0.073 | 0.345 | 2.091 | 0.929 | 2.286 | 8.43 | 24.972 | 0 |
| Seed shape | 0.001 | 0.017 | 0.042 | 0.081 | 0.081 | 0.111 | 0.154 | 0.185 | 0 |
| Rooting depth | 0.032 | 0.057 | 0.256 | 0.542 | 0.417 | 0.701 | 1.47 | 2.511 | 0 |
| Flower duration | 1 | 2 | 2.446 | 3.592 | 3 | 4 | 6 | 12 | 2 |
| Number of clonal growth organs | 1 | 1 | 1 | 2.061 | 2 | 3 | 4 | 5 | 21 |
| Number of bud bank levels | 1 | 2 | 4 | 3.718 | 4 | 4 | 4.25 | 5 | 2 |
| Ratio between number of aboveground vs.  belowground bud bank levels | -1 | -0.167 | 0.5 | 0.441 | 0.5 | 0.5 | 1 | 1 | 2 |

Tab. S4 Data summary for categorical traits.

| **Trait** | **Level** | **Number of species** |
| --- | --- | --- |
| Legume | TRUE | 22 |
|  | FALSE | 196 |
| Maximum lateral spread | <0.01 | 57 |
|  | 0.01-0.25 | 94 |
|  | >0.25 | 39 |
|  | NA | 28 |
| Maximum clonal multiplication rate | <1 | 9 |
|  | 1 | 48 |
|  | 2-10 | 117 |
|  | >10 | 11 |
|  | NA | 33 |
| Mycorrhizal status | facultative | 42 |
|  | obligate | 156 |
|  | non-mycorrhizal | 6 |
|  | NA | 14 |

Tab. S5 Aggregation of original land cover classes from EEA / Copernicus programme (2020) to land cover classes used in the analysis.

| **Original land cover class** | **New land cover classes** |
| --- | --- |
| Continuous urban fabric | urban |
| Discontinuous urban fabric | urban |
| Industrial or commercial units | urban |
| Road and rail networks and associated land | urban |
| Port areas | urban |
| Airports | urban |
| Mineral extraction sites | urban |
| Dump sites | urban |
| Construction sites | urban |
| Green urban areas | urban |
| Sport and leisure facilities | urban |
| Non-irrigated arable land | agriculture |
| Vineyards | agriculture |
| Fruit trees and berry plantations | agriculture |
| Pastures | seminatural |
| Complex cultivation patterns | agriculture |
| Land principally occupied by agriculture with  significant areas of natural vegetation | agriculture |
| Agro-forestry areas | forest |
| Broad-leaved forest | forest |
| Coniferous forest | forest |
| Mixed forest | forest |
| Natural grasslands | seminatural |
| Moors and heathland | seminatural |
| Transitional woodland-shrub | other |
| Beaches dunes sands | other |
| Bare rocks | other |
| Sparsely vegetated areas | other |
| Glaciers and perpetual snow | other |
| Inland marshes | other |
| Peat bogs | other |
| Salt marshes | other |
| Intertidal flats | other |
| Water courses | rivers / lakes |
| Water bodies | rivers / lakes |
| Coastal lagoons | other |
| Estuaries | other |
| Sea and ocean | other |

Tab. S6 Summary of rarity, trait distinctiveness and functional rarity measures at different spatial scales.

|  | **Rarity** | **Trait distinctiveness** | **Functional rarity** |
| --- | --- | --- | --- |
| **Local scale**  Data basis | Cover data per species in a relevé | Functional dissimilarity between species in a relevé  (Gower’s distance) | Scarcity and trait distinctiveness of all relevés per species |
| Formula | $S_{i}=exp(-N* A_{i}*ln(2))$ | $D_{i}= \frac{\sum_{j=1, j\neq i}^{N} d_{ij}}{N-1}$ | ${FR}_{i}= \frac{{D_{i}}^{'}+ {S_{i}}^{'}}{2}$ |
|  | S_i_ – scarcity of species i  N – number of species in the relevé  A_i_ – relative abundance of species i in the relevé | D_i_ – trait distinctiveness of species i  d_ij_ – functional distance between species i and j, scaled by maximum functional distance in the relevé  N – number of species in the relevé | FR_i_ – functional rarity of species i  D_i_’ – mean trait distinctiveness of species i scaled between 0 and 1  S_i_’ - mean scarcity of species i scaled between 0 and 1 |
| **Regional scale**  Data basis | Occurrence of a species in the relevés of a grid cell  (resolution: 20 km x 20 km) | Functional dissimilarity between species in a grid cell  (Gower’s distance; resolution: 20 km x 20 km) | Restrictedness and trait distinctiveness of all grid cells per species |
| Formula | $R_{i}=1- \frac{K_{i}}{K_{tot}}$ | $D_{i}= \frac{\sum_{j=1, j\neq i}^{N} d_{ij}}{N-1}$ | ${FR}_{i}= \frac{{D_{i}}^{'}+ {R_{i}}^{'}}{2}$ |
|  | R_i_ – restrictedness of species i  K_i_ – number of relevés in the grid cell where species i occurs  K_tot_ – total number of relevés in the grid cell | D_i_ – trait distinctiveness of species i  d_ij_ – functional distance between species i and j, scaled by maximum functional distance in the grid cell  N – number of species in the grid cell | FR_i_ – functional rarity of species i  D_i_’ – mean trait distinctiveness of species i scaled between 0 and 1  R_i_’ - mean restrictedness of species i scaled between 0 and 1 |
| **National scale**  Data basis | Occurrence of a species in a grid cell  (resolution: 20 km x 20 km) | Functional dissimilarity between species in the dataset  (Gower’s distance) | Restrictedness and trait distinctiveness per species |
| Formula | $R_{i}=1- \frac{K_{i}}{K_{tot}}$ | $D_{i}= \frac{\sum_{j=1, j\neq i}^{N} d_{ij}}{N-1}$ | ${FR}_{i}= \frac{{D_{i}}^{'}+ {R_{i}}^{'}}{2}$ |
|  | R_i_ – restrictedness of species i  K_i_ – number of grid cells where species i occurs  K_tot_ – total number of grid cells in the dataset | D_i_ – trait distinctiveness of species i  d_ij_ – functional distance between species i and j, scaled by maximum functional distance in the dataset  N – number of species in the dataset | FR_i_ – functional rarity of species i  D_i_’ – trait distinctiveness of species i scaled between 0 and 1  R_i_’ – restrictedness of species i scaled between 0 and 1 |

Tab. S7 Mean rarity, trait distinctiveness and functional rarity per species at different spatial scales.

Please find the table attached as .xlsx-file “Tab_S7_Walther_et_al.xlsx”

Tab. S8 Model parameters for boosted regression trees of species richness, number of functionally rare (FR) species and its standardized effect size (SES) per relevé.

|  | **Species richness** | **Number of FR species** | **SES of FR species** |
| --- | --- | --- | --- |
| Learning rate | 0.05 | 0.05 | 0.05 |
| Tree complexity | 10 | 10 | 10 |
| Bag fraction | 0.9 | 0.9 | 0.9 |
| Number of trees | 3150 | 1450 | 2150 |
| Number of variables in final model | 9 | 10 | 12 |

Tab. S9 Proportion of gap-filled values used for calculation of species mean trait values per species-trait combination for all 218 species included in the analyses.

| **Accepted species name** | **Specific leaf area** | **Leaf dry matter content** | **Leaf N per area** | **Plant height** | **Seed mass** | **Rooting depth** |
| --- | --- | --- | --- | --- | --- | --- |
| *Achillea millefolium* aggr*.* | 0 | 0 | 0 | 0 | 0 | 0 |
| *Achillea ptarmica* | 0 | 0 | 0 | 0 | 0 | 0,333 |
| *Aegopodium podagraria* | 0 | 0 | 0 | 0 | 0 | 0 |
| *Agrimonia eupatoria* | 0 | 0 | 0,666 | 0 | 0 | 0,333 |
| *Agrostis capillaris* | 0 | 0 | 0 | 0 | 0 | 0,333 |
| *Agrostis gigantea* | 0 | 0 | 0,333 | 0 | 0 | 0,666 |
| *Agrostis stolonifera* | 0 | 0 | 0 | 0 | 0 | 0,333 |
| *Ajuga reptans* | 0 | 0 | 0 | 0 | 0 | 0 |
| *Alchemilla monticola* | 0 | 0 | 1 | 0 | 0,333 | 0,666 |
| *Alchemilla xanthochlora* | 0 | 0 | 0 | 0 | 0 | 1 |
| *Allium scorodoprasum* | 0 | 0 | 1 | 0 | 0 | 1 |
| *Allium vineale* | 0 | 0,333 | 1 | 0 | 0 | 0 |
| *Alopecurus pratensis* | 0 | 0 | 0 | 0 | 0 | 0 |
| *Anemone nemorosa* | 0 | 0,333 | 0,666 | 0 | 0 | 0,666 |
| *Angelica sylvestris* | 0 | 0 | 0 | 0 | 0 | 0 |
| *Anisantha sterilis* | 0 | 0 | 1 | 0 | 0 | 0,666 |
| *Anthoxanthum odoratum* aggr*.* | 0 | 0 | 0 | 0 | 0 | 0 |
| *Anthriscus sylvestris* | 0 | 0 | 0 | 0 | 0 | 0,666 |
| *Anthyllis vulneraria* | 0 | 0 | 0,666 | 0 | 0 | 0 |
| *Arenaria serpyllifolia* | 0 | 0 | 1 | 0 | 0 | 0,333 |
| *Argentina anserina* | 0 | 0 | 0 | 0 | 0 | 0 |
| *Armeria maritima* | 0 | 0 | 0,333 | 0 | 0 | 0 |
| *Arrhenatherum elatius* | 0 | 0 | 0 | 0 | 0 | 0 |
| *Artemisia campestris* | 0 | 0 | 0,666 | 0 | 0 | 1 |
| *Artemisia vulgaris* | 0 | 0 | 0 | 0 | 0 | 1 |
| *Avenula pubescens* | 0 | 0 | 0 | 0 | 0 | 0 |
| *Bellis perennis* | 0 | 0 | 0 | 0 | 0 | 0,666 |
| *Bistorta officinalis* | 0 | 0 | 0 | 0 | 0 | 0 |
| *Brachypodium pinnatum* | 0 | 0 | 0,666 | 0 | 0 | 0,333 |
| *Briza media* | 0 | 0 | 0,333 | 0 | 0 | 0 |
| *Bromopsis erecta* | 0 | 0 | 0 | 0 | 0 | 0 |
| *Bromopsis inermis* | 0 | 0 | 0 | 0 | 0 | 0,666 |
| *Bromus hordeaceus* | 0 | 0 | 0 | 0 | 0 | 0,333 |
| *Bromus racemosus* | 0,333 | 1 | 1 | 0 | 0 | 0 |
| *Calamagrostis epigejos* | 0 | 0 | 0 | 0 | 0,333 | 1 |
| *Campanula glomerata* | 0 | 0 | 0,333 | 0 | 0 | 0 |
| *Campanula patula aggr.* | 0 | 0 | 0 | 0 | 0 | 0,333 |
| *Campanula rapunculoides* | 0 | 0 | 1 | 0 | 0 | 1 |
| *Campanula rapunculus* | 0 | 0 | 1 | 0 | 0,333 | 0,333 |
| *Campanula rotundifolia* | 0 | 0 | 0,333 | 0 | 0 | 0 |
| *Capsella bursa-pastoris* | 0 | 0 | 0 | 0 | 0 | 1 |
| *Cardamine pratensis* | 0 | 0 | 0 | 0 | 0 | 0 |
| *Carduus crispus* | 1 | 0,666 | 1 | 0 | 0 | 1 |
| *Carex acutiformis* | 0 | 0 | 0,666 | 0 | 0 | 0 |
| *Carex caryophyllea* | 0 | 0 | 1 | 0 | 0 | 0 |
| *Carex flacca* | 0 | 0 | 0 | 0 | 0 | 0 |
| *Carex hirta* | 0 | 0 | 0,666 | 0 | 0 | 0,666 |
| *Carex spicata* | 0 | 0 | 1 | 0 | 0 | 0,666 |
| *Carum carvi* | 0 | 0 | 0 | 0 | 0 | 0,333 |
| *Centaurea jacea* | 0 | 0 | 0 | 0 | 0 | 0 |
| *Centaurea scabiosa* | 0 | 0 | 0,666 | 0 | 0 | 0 |
| *Cerastium arvense* | 0 | 0 | 0 | 0 | 0 | 0,666 |
| *Cerastium fontanum* subsp*. vulgare* | 0 | 0 | 0,666 | 0 | 0 | 0 |
| *Cerastium semidecandrum* | 0 | 0,666 | 1 | 0 | 0 | 0,666 |
| *Cichorium intybus* | 0 | 0 | 0 | 0 | 0 | 0 |
| *Cirsium acaulon* | 0 | 0 | 1 | 0 | 0 | 0 |
| *Cirsium arvense* | 0 | 0 | 0 | 0 | 0 | 0 |
| *Cirsium oleraceum* | 0 | 0 | 0 | 0 | 0 | 0 |
| *Cirsium palustre* | 0 | 1 | 0,333 | 0 | 0 | 0 |
| *Cirsium vulgare* | 0 | 0 | 1 | 0 | 0 | 0,666 |
| *Clinopodium vulgare* | 0 | 0 | 1 | 0 | 0 | 1 |
| *Colchicum autumnale* | 0 | 0 | 1 | 0 | 0 | 0 |
| *Convolvulus arvensis* | 0 | 0 | 0 | 0 | 0 | 0,333 |
| *Crepis biennis* | 0 | 0 | 0 | 0 | 0 | 0,333 |
| *Crepis capillaris* | 0 | 0 | 1 | 0 | 0 | 0,666 |
| *Cruciata laevipes* | 0 | 0 | 1 | 0 | 0 | 0,666 |
| *Cynosurus cristatus* | 0 | 0 | 0 | 0 | 0 | 0,333 |
| *Dactylis glomerata* | 0 | 0 | 0 | 0 | 0 | 0 |
| *Danthonia decumbens* | 0 | 0 | 1 | 0 | 0 | 0,333 |
| *Daucus carota* | 0 | 0 | 0 | 0 | 0 | 0 |
| *Deschampsia cespitosa* aggr*.* | 0 | 0 | 0 | 0 | 0 | 0 |
| *Dianthus carthusianorum* aggr*.* | 0 | 1 | 1 | 0 | 0 | 0,333 |
| *Dianthus deltoides* | 0 | 0 | 0,666 | 0 | 0 | 0 |
| *Draba verna aggr.* | 0 | 0 | 1 | 0 | 0 | 0,666 |
| *Elytrigia repens aggr.* | 0 | 0 | 0 | 0 | 0 | 0,333 |
| *Equisetum arvense* | 0 | 0 | 0 | 0 | 1 | 0 |
| *Equisetum palustre* | 0 | 0,333 | 0,666 | 0,666 | 1 | 0,666 |
| *Eryngium campestre* | 0 | 0 | 0,333 | 0 | 0 | 0,333 |
| *Euphorbia cyparissias* | 0 | 0 | 1 | 0 | 0 | 0 |
| *Euphorbia esula* | 0 | 0 | 0 | 0 | 0 | 1 |
| *Falcaria vulgaris* | 0 | 0 | 1 | 0 | 0,333 | 1 |
| *Festuca ovina* | 0 | 0 | 0 | 0 | 0 | 0,333 |
| *Festuca rubra* aggr*.* | 0 | 0 | 0 | 0 | 0 | 0 |
| *Ficaria fascicularis* | 0 | 0,333 | 1 | 0 | 0 | 0 |
| *Ficaria verna* | 0 | 0,333 | 0,333 | 0 | 0 | 0 |
| *Filipendula ulmaria* | 0 | 0 | 0 | 0 | 0 | 0 |
| *Filipendula vulgaris* | 0 | 0 | 0,333 | 0 | 0 | 0 |
| *Fragaria vesca* | 0 | 0 | 0 | 0 | 0 | 0,666 |
| *Fragaria viridis* | 0 | 0 | 0,333 | 0 | 0,666 | 0,666 |
| *Galium aparine* | 0 | 0 | 0,333 | 0 | 0 | 0,666 |
| *Galium boreale* | 0 | 0 | 0 | 0 | 0 | 0 |
| *Galium mollugo* aggr*.* | 0 | 0 | 0 | 0 | 0 | 0,666 |
| *Galium pumilum* | 0 | 0 | 1 | 0 | 0,333 | 1 |
| *Galium uliginosum* | 0 | 0 | 1 | 0 | 0,333 | 1 |
| *Galium verum* | 0 | 0 | 0 | 0 | 0 | 0 |
| *Geranium dissectum* | 0 | 0 | 1 | 0 | 0 | 1 |
| *Geranium molle* | 0 | 0 | 1 | 0 | 0 | 0,666 |
| *Geranium pratense* | 0 | 0 | 0 | 0 | 0 | 0 |
| *Geranium pusillum* | 0 | 0 | 1 | 0 | 0 | 0,666 |
| *Geranium sylvaticum* aggr*.* | 0 | 0 | 0 | 0 | 0 | 0 |
| *Geum rivale* | 0 | 0 | 0 | 0 | 0 | 0 |
| *Glechoma hederacea* | 0 | 0 | 0 | 0 | 0 | 1 |
| *Helictochloa pratensis* | 0 | 0 | 0,666 | 0 | 0 | 0 |
| *Heracleum sphondylium* | 0 | 0 | 0 | 0 | 0 | 0 |
| *Hieracium umbellatum* | 0 | 0 | 0,666 | 0 | 0 | 0 |
| *Holcus lanatus* | 0 | 0 | 0 | 0 | 0 | 0 |
| *Holcus mollis* | 0 | 0,333 | 0,666 | 0 | 0 | 0,666 |
| *Hypericum maculatum* aggr*.* | 0 | 0 | 0,666 | 0 | 0 | 0 |
| *Hypericum perforatum* | 0 | 0 | 0,666 | 0 | 0 | 0 |
| *Hypochaeris radicata* | 0 | 0 | 0 | 0 | 0 | 0 |
| *Jacobaea vulgaris* | 0 | 0 | 1 | 0 | 0 | 0 |
| *Knautia arvensis* | 0 | 0 | 0 | 0 | 0 | 0 |
| *Koeleria pyramidata* | 0 | 0 | 0,333 | 0 | 1 | 0,666 |
| *Lamium album* | 0 | 0 | 1 | 0 | 0 | 1 |
| *Lathyrus linifolius* | 0 | 0,333 | 1 | 0 | 0 | 1 |
| *Lathyrus pratensis* | 0 | 0 | 0 | 0 | 0 | 0 |
| *Leontodon hispidus* | 0 | 0 | 0 | 0 | 0 | 0 |
| *Leucanthemum vulgare* aggr*.* | 0 | 0 | 0 | 0 | 0 | 0 |
| *Linaria vulgaris* | 0 | 0 | 0,666 | 0 | 0 | 1 |
| *Linum catharticum* | 0 | 0 | 0 | 0 | 0 | 0 |
| *Lolium multiflorum* | 0 | 0 | 0 | 0 | 0 | 0,666 |
| *Lolium perenne* | 0 | 0 | 0 | 0 | 0 | 0 |
| *Lotus corniculatus* | 0 | 0 | 0 | 0 | 0 | 0 |
| *Lotus pedunculatus* | 0 | 0 | 0 | 0 | 0 | 0 |
| *Luzula campestris* aggr*.* | 0 | 0 | 0 | 0 | 0 | 0,333 |
| *Lysimachia nummularia* | 0 | 0 | 1 | 0 | 0,333 | 0,333 |
| *Lythrum salicaria* | 0 | 0 | 0 | 0 | 0 | 0,333 |
| *Malva moschata* | 0 | 0 | 1 | 0 | 0,333 | 1 |
| *Medicago falcata* | 0 | 0 | 1 | 0,333 | 0 | 0 |
| *Medicago lupulina* | 0 | 0 | 0 | 0 | 0 | 0 |
| *Medicago sativa* aggr*.* | 0 | 0 | 0 | 0 | 0 | 0 |
| *Myosotis arvensis* | 0 | 0,333 | 1 | 0 | 0 | 1 |
| *Myosotis ramosissima* | 0 | 0 | 1 | 0 | 0 | 0,666 |
| *Myosotis scorpioides* aggr*.* | 0 | 0 | 1 | 0 | 0 | 0,666 |
| *Ochlopoa annua* | 0 | 0 | 0 | 0 | 0 | 0,666 |
| *Onobrychis viciifolia* | 0 | 0 | 0 | 0 | 0 | 0 |
| *Origanum vulgare* | 0 | 0 | 0 | 0 | 0 | 0,666 |
| *Pastinaca sativa* | 0 | 0 | 0 | 0 | 0 | 0 |
| *Persicaria amphibia* | 0 | 0 | 1 | 0 | 0 | 0,333 |
| *Phalaroides arundinacea* | 0 | 0 | 0 | 0 | 0 | 0,666 |
| *Phleum pratense* | 0 | 0 | 0 | 0 | 0 | 0 |
| *Phyteuma nigrum* | 0 | 0 | 1 | 0,666 | 0,333 | 0 |
| *Picris hieracioides* | 0 | 0 | 0 | 0 | 0 | 0,333 |
| *Pilosella officinarum* | 0 | 0 | 0 | 0 | 0 | 0 |
| *Pimpinella major* | 0 | 0 | 0 | 0 | 0 | 0,666 |
| *Pimpinella saxifraga* | 0 | 0 | 1 | 0 | 0 | 0 |
| *Plantago lanceolata* | 0 | 0 | 0 | 0 | 0 | 0 |
| *Plantago major* | 0 | 0 | 0 | 0 | 0 | 0,333 |
| *Plantago media* | 0 | 0 | 0 | 0 | 0 | 0 |
| *Poa pratensis* aggr*.* | 0 | 0 | 0 | 0 | 0 | 0 |
| *Poa trivialis* | 0 | 0 | 0 | 0 | 0 | 0 |
| *Polygala vulgaris* | 0 | 0 | 0 | 0 | 0 | 0,666 |
| *Potentilla argentea* | 0 | 0 | 1 | 0 | 0,666 | 0 |
| *Potentilla erecta* | 0 | 0 | 0,333 | 0 | 0 | 0 |
| *Potentilla reptans* | 0 | 0 | 0 | 0 | 0 | 0 |
| *Potentilla tabernaemontani* | 0 | 0 | 1 | 0 | 0 | 0,333 |
| *Primula veris* | 0 | 0 | 0,333 | 0 | 0 | 0 |
| *Prunella vulgaris* | 0 | 0 | 0 | 0 | 0 | 0 |
| *Ranunculus acris* aggr*.* | 0 | 0 | 0 | 0 | 0 | 0 |
| *Ranunculus bulbosus* | 0 | 0 | 0 | 0 | 0 | 0,333 |
| *Ranunculus polyanthemos* | 0 | 0 | 0,333 | 0 | 0,666 | 0,666 |
| *Ranunculus repens* | 0 | 0 | 0 | 0 | 0 | 0,333 |
| *Rhinanthus alectorolophus* | 0 | 0 | 0,333 | 0 | 0 | 0,666 |
| *Rhinanthus angustifolius* | 0 | 0 | 0,333 | 0 | 0 | 1 |
| *Rhinanthus minor* | 0 | 0,333 | 0 | 0 | 0 | 0 |
| *Rumex acetosa* | 0 | 0 | 0 | 0 | 0 | 0 |
| *Rumex acetosella* | 0 | 0 | 0 | 0 | 0 | 1 |
| *Rumex crispus* | 0 | 0 | 1 | 0 | 0 | 0 |
| *Rumex obtusifolius* | 0 | 0 | 0 | 0 | 0 | 0 |
| *Rumex thyrsiflorus* | 0 | 0 | 1 | 0 | 0 | 0,333 |
| *Salvia pratensis* | 0 | 0 | 1 | 0 | 0 | 0 |
| *Sanguisorba minor* aggr*.* | 0 | 0 | 0 | 0 | 0 | 0 |
| *Sanguisorba officinalis* | 0 | 0 | 0 | 0 | 0 | 0 |
| *Saxifraga granulata* | 0 | 0,666 | 1 | 0 | 0 | 0,666 |
| *Scabiosa columbaria* aggr*.* | 0 | 0 | 0,666 | 0 | 0 | 0 |
| *Schedonorus arundinaceus* | 0 | 0 | 0 | 0 | 0 | 0 |
| *Schedonorus pratensis* | 0 | 0 | 0 | 0 | 0 | 0 |
| *Scorzoneroides autumnalis* | 0 | 0 | 0 | 0 | 0 | 0 |
| *Securigera varia* | 0 | 0 | 1 | 0 | 0 | 1 |
| *Selinum carvifolia* | 0 | 0 | 1 | 0 | 0 | 0 |
| *Silaum silaus* | 0 | 0,333 | 1 | 0 | 0 | 0 |
| *Silene dioica* | 0 | 0 | 0 | 0 | 0 | 0,666 |
| *Silene flos-cuculi* | 0 | 0 | 1 | 0 | 0 | 0,333 |
| *Silene latifolia* | 0 | 0 | 1 | 0 | 0 | 0,666 |
| *Silene vulgaris* | 0 | 0 | 0 | 0 | 0 | 0,666 |
| *Solidago virgaurea* | 0 | 0 | 0 | 0 | 0 | 1 |
| *Stachys officinalis* | 0 | 0 | 0 | 0 | 0 | 0 |
| *Stellaria graminea* | 0 | 0 | 1 | 0 | 0 | 0,333 |
| *Stellaria media* | 0 | 0,333 | 0 | 0 | 0 | 0,666 |
| *Succisa pratensis* | 0 | 0 | 0 | 0 | 0 | 0 |
| *Symphytum officinale* | 0 | 1 | 1 | 0 | 0 | 1 |
| *Tanacetum vulgare* | 0 | 0 | 0 | 0 | 0 | 1 |
| *Taraxacum* sect*. Taraxacum* | 0 | 0 | 0 | 0 | 0 | 0,333 |
| *Torilis japonica* | 0 | 0 | 1 | 0 | 0 | 1 |
| *Tragopogon pratensis* | 0 | 0 | 0 | 0 | 0 | 0,666 |
| *Trifolium arvense* | 0 | 0 | 1 | 0 | 0 | 0,666 |
| *Trifolium campestre* | 0 | 0 | 0 | 0 | 0 | 0 |
| *Trifolium dubium* | 0 | 0 | 0 | 0 | 0 | 0,666 |
| *Trifolium hybridum* | 0 | 0 | 0 | 0 | 0 | 0,666 |
| *Trifolium medium* | 0 | 0,666 | 1 | 0 | 0 | 0 |
| *Trifolium pratense* | 0 | 0 | 0 | 0 | 0 | 0 |
| *Trifolium repens* | 0 | 0 | 0 | 0 | 0 | 0,333 |
| *Tripleurospermum maritimum* aggr*.* | 0 | 0 | 0,666 | 0 | 0 | 1 |
| *Trisetum flavescens* | 0 | 0 | 0 | 0 | 0 | 0 |
| *Urtica dioica* | 0 | 0 | 0 | 0 | 0 | 1 |
| *Valeriana officinalis* aggr*.* | 0 | 0 | 1 | 0 | 0 | 0,666 |
| *Valerianella locusta* | 0,333 | 0,666 | 1 | 0 | 0 | 0,666 |
| *Veronica arvensis* | 0 | 0 | 1 | 0 | 0 | 0,666 |
| *Veronica chamaedrys* aggr*.* | 0 | 0 | 0 | 0 | 0 | 0 |
| *Veronica officinalis* | 0 | 0,666 | 0,666 | 0 | 0 | 0 |
| *Veronica serpyllifolia* | 0 | 0,666 | 1 | 0 | 0 | 1 |
| *Vicia cracca* | 0 | 0 | 0 | 0 | 0 | 0,666 |
| *Vicia hirsuta* | 0 | 0 | 1 | 0 | 0 | 1 |
| *Vicia sativa* | 0 | 0 | 0,333 | 0 | 0 | 0,666 |
| *Vicia sepium* | 0 | 0 | 0 | 0 | 0 | 0 |
| *Vicia tetrasperma* | 0 | 0 | 1 | 0 | 0 | 1 |
| *Viola canina* | 0 | 0 | 0,333 | 0 | 0 | 0 |
| *Viola hirta* | 0 | 0 | 1 | 0 | 0 | 0 |

# References

EEA / Copernicus programme (2020) *Corine Land Cover (CLC) 2018, Version 2020_20u1*. <https://land.copernicus.eu/pan-european/corine-land-cover>
