# Supplementary material for: Functional rarity of plants in German hay meadows — Patterns on the species level and mismatches with community species richness
Source: Ecol Evol. 2022 Oct 1;12(10):e9375. doi: 10.1002/ece3.9375 (PMC9526122; doi:10.1002/ece3.9375)
Supplement: Supplementary file 3 — Appendix S1 [file ECE3-12-e9375-s006.docx]

# Appendix S1. Trait data cleaning and aggregation

For trait data from the TRY database (version 5.0; (Kattge et al., 2020)), observations that i) were marked as duplicates, ii) showed a high error probability (TRY internal parameter “error risk” > 3) or iii) that did not refer to single trait measurements or mean trait values were excluded from the analysis. Furthermore, traits of lower levels (in the case of specific leaf area (SLA) e.g., “specific leaf area: petiole included” and “specific leaf area: undefined if petiole is in- or excluded”) were merged at higher levels (e.g., “specific leaf area”). Due to low coverage, trait data for rooting depth in TRY were complemented by data from Kutschera et al. (1982) and Kutschera et al. (1992).

For the continuous traits from the TRY database (SLA, leaf dry matter content, leaf N per area, plant height, seed mass, seed length, seed width, seed thickness and rooting depth), species mean trait values were calculated in two consecutive steps: i) For each species-trait combination with at least three observations, species means were calculated using only measured trait data. ii) For each species-trait combination with less than three observations, gap-filled trait data (procedure described in Schrodt et al. (2015), applied to TRY version 3.0) were used to complement measured trait data and to calculate species means. The relative weight of gap-filled data was reduced as follows: When two observations were available, all gap-filled data were averaged to produce one value fully based on gap-filled data. The two observations were combined with the mean gap-filled value to calculate the final species mean trait value (weight of measured : gap-filled data = 2:1). When one observation was available, all gap-filled data were split into two equally sized groups. For each group of gap-filled data an average gap-filled trait value was calculated. The observation was combined with these two average gap-filled values to calculate the final species mean trait value (weight of measured : gap-filled data = 1:2). When no observations were available, the final species mean trait value is based on gap-filled trait data only. Species with less than three observations for any of these traits (measured and gap-filled combined) were excluded from the further analyses. For an overview on the percentage of gap-filled data used per species-trait combination see Tab. S9.

Mean values of seed length, width and thickness were only used to calculate seed shape per species. Seed shape represents the variance of seed length, width, and thickness, each scaled by seed length as described in Thompson et al. (1993). For two ordinal scaled traits (maximum lateral spread and maximum clonal reproduction rate), maximum values were derived from CLO-PLA (Klimešová et al., 2017). The number of bud bank levels was calculated from presence of regenerative buds per species at each level. The ratio between the number of above- and belowground bud bank levels was calculated as the fraction of aboveground bud bank levels minus the fraction of belowground bud bank levels occupied by a species, independent from the number of buds per level. Data on the number of clonal growth organs (e.g., bulb, epigeogenous rhizome) were extracted from CLO-PLA (Klimešová et al., 2017) without further processing (Tab. 1). Information on species-specific mycorrhizal associations (Guerrero‐Ramírez et al., 2020) were used to derive the mycorrhizal status of a species. Species that are sometimes, but not necessarily associated with mycorrhizal fungi are classified as “facultative mycorrhizal”. Species that are always associated with mycorrhizal fungi are classified as “obligate mycorrhizal”. Species that are never associated with mycorrhizal fungi are classified as “non-mycorrhizal” (Tab. 1).

# References

Guerrero‐Ramírez, N. R., Mommer, L., Freschet, G. T., Iversen, C. M., McCormack, M. L., Kattge, J., Poorter, H. et al. (2020) Global root traits (GRooT) database. *Global Ecology and Biogeography,* 00**,** 1-13. <https://doi.org/10.1111/geb.13179>

Kattge, J., Bonisch, G., Diaz, S., Lavorel, S., Prentice, I. C., Leadley, P., Tautenhahn, S. et al. (2020) TRY plant trait database - enhanced coverage and open access. *Global change biology,* 26**,** 119-188. <https://doi.org/10.1111/gcb.14904>

Klimešová, J., Danihelka, J., Chrtek, J., de Bello, F. & Herben, T. (2017) CLO‐PLA: A database of clonal and bud‐bank traits of the Central European flora. *Ecology,* 98**,** 1179. <https://doi.org/10.1002/ecy.1745>

Kutschera, L., Lichtenegger, E. & Sobotik, M. (1982) *Root atlas of Central European grassland plants. Monocotyledoneae* (German). Stuttgart, New York: Gustav Fischer Verlag.

Kutschera, L., Lichtenegger, E. & Sobotik, M. (1992) *Root atlas of Central European grassland plants. Pteridophyta and Dicotyledoneae (Magnoliopsida): Morphology, Anatomy, Ecology, Distribution, Sociology, Economy* (German). Stuttgart, Jena, New York: Gustav Fischer Verlag.

Schrodt, F., Kattge, J., Shan, H., Fazayeli, F., Joswig, J., Banerjee, A., Reichstein, M. et al. (2015) BHPMF–a hierarchical B ayesian approach to gap‐filling and trait prediction for macroecology and functional biogeography. *Global Ecology and Biogeography,* 24**,** 1510-1521. <https://doi.org/10.1111/geb.12335>

Thompson, K., Band, S. R. & Hodgson, J. G. (1993) Seed Size and Shape Predict Persistence in Soil. *Functional Ecology,* 7**,** 236-241. <https://doi.org/10.2307/2389893>
