# Supplementary material for: Functional rarity of plants in German hay meadows — Patterns on the species level and mismatches with community species richness
Source: Ecol Evol. 2022 Oct 1;12(10):e9375. doi: 10.1002/ece3.9375 (PMC9526122; doi:10.1002/ece3.9375)
Supplement: Supplementary file 4 — Appendix S2 [file ECE3-12-e9375-s003.docx]

# Appendix S2. Additional information on boosted regression trees

Boosted regression trees (BRTs) combine decision trees with gradient boosting (Elith et al., 2008). While decision trees can deal with different types of variables, missing values, non-linear relationships and are easy to interpret (De'ath & Fabricius, 2000), their predictive performance is limited. In BRTs, boosting improves the overall model accuracy by iteratively fitting many decision trees, with each tree reducing the predictive deviance of the previous one (i.e., stagewise reduction of residuals; a more detailed description how BRTs work is given in Elith et al. (2008)). Interpretation of BRTs is facilitated by returning the proportion of deviance explained by the final model, the relative importance of each predictor variable in the final model and partial dependence plots that show the modelled relationship of the response to each predictor variable.

We used BRTs to model species richness, the the number of functionally rare species and its standardized effect size per relevé depending on 12 environmental variables. To find the model with the lowest predictive deviance, we fitted BRTs with a maximum number of 25 000 trees and all possible combinations of the model parameters learning rate (0.2, 0.1, 0.05, 0.01, 0.005, 0.001), tree complexity (1 to 10) and bag fraction (0.1 to 0.9 in steps of 0.1). Parameter combinations with more than 25 000 trees were excluded from the search due to high computation effort.

# References

De'ath, G. & Fabricius, K. E. (2000) Classification and regression trees: A powerful yet simple technique for ecological data analysis. *Ecology,* 81**,** 3178-3192. [https://doi.org/10.1890/0012-9658(2000)081[3178:Cartap]2.0.Co;2](https://doi.org/10.1890/0012-9658(2000)081%5b3178:Cartap%5d2.0.Co;2)

Elith, J., Leathwick, J. R. & Hastie, T. (2008) A working guide to boosted regression trees. *Journal of Animal Ecology,* 77**,** 802-813. <https://doi.org/10.1111/j.1365-2656.2008.01390.x>
