# Supplementary material for: Functional rarity of plants in German hay meadows — Patterns on the species level and mismatches with community species richness
Source: Ecol Evol. 2022 Oct 1;12(10):e9375. doi: 10.1002/ece3.9375 (PMC9526122; doi:10.1002/ece3.9375)
Supplement: Supplementary file 5 — Appendix S3 [file ECE3-12-e9375-s005.docx]

# Appendix S3. References of trait data from TRY used in the analysis

Adler, P. B., Salguero-Gomez, R., Compagnoni, A., Hsu, J. S., Ray-Mukherjee, J., Mbeau-Ache, C. & Franco, M. (2014) Functional traits explain variation in plant life history strategies. PNAS, 111, 740-5. <https://doi.org/10.1073/pnas.1315179111>

Atkin, O. K., Bloomfield, K. J., Reich, P. B., Tjoelker, M. G., Asner, G. P., Bonal, D., Bönisch, G. et al. (2015) Global variability in leaf respiration in relation to climate, plant functional types and leaf traits. New phytologist, 206, 614-36. <https://doi.org/10.1111/nph.13253>

Atkin, O. K., Westbeek, M. H., Cambridge, M. L., Lambers, H. & Pons, T. L. (1997) Leaf respiration in light and darkness (a comparison of slow-and fast-growing Poa species). Plant Physiology, 113, 961-965. <https://doi.org/10.1104/pp.113.3.961>

Baruch, Z. & Goldstein, G. (1999) Leaf construction cost, nutrient concentration, and net CO 2 assimilation of native and invasive species in Hawaii. Oecologia, 121, 183-192.

Beckmann, M., Hock, M., Bruelheide, H. & Erfmeier, A. (2012) The role of UV-B radiation in the invasion of Hieracium pilosella-A comparison of German and New Zealand plants. Environmental and Experimental Botany, 75, 173-180. <https://doi.org/10.1016/j.envexpbot.2011.09.010>

Belluau, M. & Shipley, B. (2018) Linking hard and soft traits: Physiology, morphology and anatomy interact to determine habitat affinities to soil water availability in herbaceous dicots. Plos one, 13, e0193130. <https://doi.org/10.1371/journal.pone.0193130>

Bragazza, L. (2009) Conservation priority of Italian Alpine habitats: a floristic approach based on potential distribution of vascular plant species. Biodiversity and conservation, 18, 2823-2835. <https://doi.org/10.1007/s10531-009-9609-3>

Burrascano, S., Copiz, R., Del Vico, E., Fagiani, S., Giarrizzo, E., Mei, M., Mortelliti, A. et al. (2015) Wild boar rooting intensity determines shifts in understorey composition and functional traits. Community Ecology, 16, 244-253. <https://doi.org/10.1556/168.2015.16.2.12>

Campbell, C., Atkinson, L., Zaragoza-Castells, J., Lundmark, M., Atkin, O. & Hurry, V. (2007) Acclimation of photosynthesis and respiration is asynchronous in response to changes in temperature regardless of plant functional group. New phytologist, 176, 375-389. <https://doi.org/10.1111/j.1469-8137.2007.02183.x>

Campetella, G., Botta-Dukat, Z., Wellstein, C., Canullo, R., Gatto, S., Chelli, S., Mucina, L. et al. (2011) Patterns of plant trait-environment relationships along a forest succession chronosequence. Agriculture Ecosystems & Environment, 145, 38-48. <https://doi.org/10.1016/j.agee.2011.06.025>

Ciccarelli, D. (2015) Mediterranean coastal dune vegetation: are disturbance and stress the key selective forces that drive the psammophilous succession? Estuarine, Coastal and Shelf Science, 165, 247-253. <https://doi.org/10.1016/j.ecss.2015.05.023>

Ciocarlan, V. (2009) The illustrated Flora of Romania. Pteridophyta et Spermatopyta (Romanian). Editura Ceres.

Cornelissen, J. H. C. (1996) An experimental comparison of leaf decomposition rates in a wide range of temperate plant species and types. Journal of Ecology, 84, 573-582. <https://doi.org/10.2307/2261479>

Cornelissen, J. H. C., Quested, H. M., Gwynn-Jones, D., Van Logtestijn, R. S. P., De Beus, M. A. H., Kondratchuk, A., Callaghan, T. V. et al. (2004) Leaf digestibility and litter decomposability are related in a wide range of subarctic plant species and types. Functional Ecology, 18, 779-786. <https://doi.org/10.1111/j.0269-8463.2004.00900.x>

Craine, J. M., Nippert, J. B., Towne, E. G., Tucker, S., Kembel, S. W., Skibbe, A. & McLauchlan, K. K. (2011) Functional consequences of climate change-induced plant species loss in a tallgrass prairie. Oecologia, 165, 1109-17. <https://doi.org/10.1007/s00442-011-1938-8>

Dalke, I. V., Novakovskiy, A. B., Maslova, S. P. & Dubrovskiy, Y. A. (2018) Morphological and functional traits of herbaceous plants with different functional types in the European Northeast. Plant Ecology, 219, 1295-1305. <https://doi.org/10.1007/s11258-018-0879-2>

de Vries, F. T. & Bardgett, R. D. (2016) Plant community controls on short-term ecosystem nitrogen retention. New phytologist, 210, 861-74. <https://doi.org/10.1111/nph.13832>

Diaz, S., Hodgson, J. G., Thompson, K., Cabido, M., Cornelissen, J. H. C., Jalili, A., Montserrat-Marti, G. et al. (2004) The plant traits that drive ecosystems: Evidence from three continents. Journal of vegetation science, 15, 295-304. <https://doi.org/10.1111/j.1654-1103.2004.tb02266.x>

Dwyer, J. M., Hobbs, R. J. & Mayfield, M. M. (2014) Specific leaf area responses to environmental gradients through space and time. Ecology, 95, 399-410. <https://doi.org/10.1890/13-0412.1>

Everwand, G., Fry, E., Eggers, T. & Manning, P. (2014) Seasonal variation in the relationship between plant traits and grassland carbon and water fluxes. Ecosystems, 17, 1095-1108.

Fan, Y., Miguez-Macho, G., Jobbágy, E. G., Jackson, R. B. & Otero-Casal, C. (2017) Hydrologic regulation of plant rooting depth. PNAS, 114, 10572-10577. <https://doi.org/10.1073/pnas.1712381114>

Fitter, A. H. & Peat, H. J. (1994) The Ecological Flora Database. Journal of Ecology, 82, 415-425. <https://doi.org/10.2307/2261309>

Fonseca, C. R., Overton, J. M., Collins, B. & Westoby, M. (2000) Shifts in trait‐combinations along rainfall and phosphorus gradients. Journal of Ecology, 88, 964-977. <https://doi.org/10.1046/j.1365-2745.2000.00506.x>

Freschet, G. T., Cornelissen, J. H., Van Logtestijn, R. S. & Aerts, R. (2010) Evidence of the ‘plant economics spectrum’in a subarctic flora. Journal of Ecology, 98, 362-373. <https://doi.org/10.1111/j.1365-2745.2009.01615.x>

Fry, E. L., Power, S. A. & Manning, P. (2014) Trait-based classification and manipulation of plant functional groups for biodiversity-ecosystem function experiments. Journal of vegetation science, 25, 248-261. <https://doi.org/10.1111/jvs.12068>

Giarrizzo, E., Burrascano, S., Chiti, T., de Bello, F., Lepš, J., Zavattero, L. & Blasi, C. (2017) Re‐visiting historical semi‐natural grasslands in the Apennines to assess patterns of changes in species composition and functional traits. Applied Vegetation Science, 20, 247-258. <https://doi.org/10.1111/avsc.12288>

Gos, P., Loucougaray, G., Colace, M.-P., Arnoldi, C., Gaucherand, S., Dumazel, D., Girard, L. et al. (2016) Relative contribution of soil, management and traits to co-variations of multiple ecosystem properties in grasslands. Oecologia, 180, 1001-1013. <https://doi.org/10.1007/s00442-016-3551-3>

Green, W. A. (2009) USDA PLANTS Compilation. Available: http://bricol.net/downloads/data/PLANTSdatabase/ [Accessed 09-02-02].

Guy, A. L., Mischkolz, J. M. & Lamb, E. G. (2013) Limited effects of simulated acidic deposition on seedling survivorship and root morphology of endemic plant taxa of the Athabasca Sand Dunes in well-watered greenhouse trials. Botany, 91, 176-181. <https://doi.org/10.1139/cjb-2012-0162>

Herz, K., Dietz, S., Haider, S., Jandt, U., Scheel, D. & Bruelheide, H. (2017) Drivers of intraspecific trait variation of grass and forb species in German meadows and pastures. Journal of vegetation science, 28, 705-716. <https://doi.org/10.1111/jvs.12534>

Herz, K., Dietz, S., Haider, S., Jandt, U., Scheel, D. & Bruelheide, H. (2017) Predicting individual plant performance in grasslands. Ecology and Evolution, 7, 8958-8965. <https://doi.org/10.1002/ece3.3393>

Iversen, C. M., McCormack, M. L., Powell, A. S., Blackwood, C. B., Freschet, G. T., Kattge, J., Roumet, C. et al. (2017) A global Fine-Root Ecology Database to address below-ground challenges in plant ecology. New phytologist, 215, 15-26. <https://doi.org/10.1111/nph.14486>

Kattge, J., Knorr, W., Raddatz, T. & Wirth, C. (2009) Quantifying photosynthetic capacity and its relationship to leaf nitrogen content for global-scale terrestrial biosphere models. Global change biology, 15, 976-991. <https://doi.org/10.1111/j.1365-2486.2008.01744.x>

Kazakou, E., Vile, D., Shipley, B., Gallet, C. & Garnier, E. (2006) Co-variations in litter decomposition, leaf traits and plant growth in species from a Mediterranean old-field succession. Functional Ecology, 20, 21-30. <https://doi.org/10.1111/j.1365-2435.2006.01080.x>

Kichenin, E., Wardle, D. A., Peltzer, D. A., Morse, C. W. & Freschet, G. T. (2013) Contrasting effects of plant inter‐and intraspecific variation on community‐level trait measures along an environmental gradient. Functional Ecology, 27, 1254-1261. <https://doi.org/10.1111/1365-2435.12116>

Kleyer, M., Bekker, R. M., Knevel, I. C., Bakker, J. P., Thompson, K., Sonnenschein, M., Poschlod, P. et al. (2008) The LEDA Traitbase: a database of life-history traits of the Northwest European flora. Journal of Ecology, 96, 1266-1274. <https://doi.org/10.1111/j.1365-2745.2008.01430.x>

Kühn, I., Durka, W. & Klotz, S. (2004) BiolFlor: a new plant-trait database as a tool for plant invasion ecology. Diversity and Distributions, 10, 363-365.

Laughlin, D. C., Leppert, J. J., Moore, M. M. & Sieg, C. H. (2010) A multi‐trait test of the leaf‐height‐seed plant strategy scheme with 133 species from a pine forest flora. Functional Ecology, 24, 493-501. <https://doi.org/10.1111/j.1365-2435.2009.01672.x>

Lhotsky, B., Csecserits, A., Kovács, B. & Botta-Dukát, Z. (2016) New plant trait records of the Hungarian flora. Acta Botanica Hungarica, 58, 397-400.

Li, Y. & Shipley, B. (2018) Community divergence and convergence along experimental gradients of stress and disturbance. Ecology, 99, 775-781. <https://doi.org/10.1002/ecy.2162>

Louault, F., Pillar, V. D., Aufrere, J., Garnier, E. & Soussana, J. F. (2005) Plant traits and functional types in response to reduced disturbance in a semi-natural grassland. Journal of vegetation science, 16, 151-160. <https://doi.org/10.1111/j.1654-1103.2005.tb02350.x>

Loveys, B., Atkinson, L. J., Sherlock, D., Roberts, R. L., Fitter, A. H. & Atkin, O. K. (2003) Thermal acclimation of leaf and root respiration: an investigation comparing inherently fast‐and slow‐growing plant species. Global change biology, 9, 895-910. <https://doi.org/10.1046/j.1365-2486.2003.00611.x>

Maire, V., Wright, I. J., Prentice, I. C., Batjes, N. H., Bhaskar, R., van Bodegom, P. M., Cornwell, W. K. et al. (2015) Global effects of soil and climate on leaf photosynthetic traits and rates. Global Ecology and Biogeography, 24, 706-717. <https://doi.org/10.1111/geb.12296>

Maire, V., Wright, I. J., Prentice, I. C., Batjes, N. H., Bhaskar, R., van Bodegom, P. M., Cornwell, W. K. et al. (2016) Data from: Global effects of soil and climate on leaf photosynthetic traits and rates. Dryad.

Manning, P., Houston, K. & Evans, T. (2009) Shifts in seed size across experimental nitrogen enrichment and plant density gradients. Basic and Applied Ecology, 10, 300-308. <https://doi.org/10.1016/j.baae.2008.08.004>

Meziane, D. & Shipley, B. (1999) Interacting determinants of specific leaf area in 22 herbaceous species: effects of irradiance and nutrient availability. Plant, Cell & Environment, 22, 447-459. <https://doi.org/10.1046/j.1365-3040.1999.00423.x>

Milla, R. & Reich, P. B. (2011) Multi-trait interactions, not phylogeny, fine-tune leaf size reduction with increasing altitude. Annals of Botany, 107, 455-465.

Moles, A. T., Falster, D. S., Leishman, M. R. & Westoby, M. (2004) Small‐seeded species produce more seeds per square metre of canopy per year, but not per individual per lifetime. Journal of Ecology, 92, 384-396. <https://doi.org/10.1111/j.0022-0477.2004.00880.x>

Moretti, M. & Legg, C. (2009) Combining plant and animal traits to assess community functional responses to disturbance. Ecography, 32, 299-309. <https://doi.org/10.1111/j.1600-0587.2008.05524.x>

Onoda, Y., Wright, I. J., Evans, J. R., Hikosaka, K., Kitajima, K., Niinemets, Ü., Poorter, H. et al. (2017) Physiological and structural tradeoffs underlying the leaf economics spectrum. New phytologist, 214, 1447-1463. <https://doi.org/10.1111/nph.14496>

Ordonez, J. C., van Bodegom, P. M., Witte, J.-P. M., Bartholomeus, R. P., van Hal, J. R. & Aerts, R. (2010) Plant strategies in relation to resource supply in mesic to wet environments: does theory mirror nature? The American Naturalist, 175, 225-239.

Paula, S., Arianoutsou, M., Kazanis, D., Tavsanoglu, Ç., Lloret, F., Buhk, C., Ojeda, F. et al. (2009) Fire-related traits for plant species of the Mediterranean Basin. Ecology, 90, 1420-1420. <https://doi.org/10.1890/08-1309.1>

Peco, B., de Pablos, I., Traba, J. & Levassor, C. (2005) The effect of grazing abandonment on species composition and functional traits: the case of dehesa grasslands. Basic and Applied Ecology, 6, 175-183. <https://doi.org/10.1016/j.baae.2005.01.002>

Prentice, I. C., Meng, T., Wang, H., Harrison, S. P., Ni, J. & Wang, G. (2011) Evidence of a universal scaling relationship for leaf CO2 drawdown along an aridity gradient. New phytologist, 190, 169-180. <https://doi.org/10.1111/j.1469-8137.2010.03579.x>

Price, C. A. & Enquist, B. J. (2007) Scaling mass and morphology in leaves: an extension of the WBE model. Ecology, 88, 1132-1141. <https://doi.org/10.1890/06-1158>

Pyankov, V. I., Kondratchuk, A. V. & Shipley, B. (1999) Leaf structure and specific leaf mass: the alpine desert plants of the Eastern Pamirs, Tadjikistan. New phytologist, 143, 131-142. <https://doi.org/10.1046/j.1469-8137.1999.00435.x>

Quested, H. M., Cornelissen, J. H. C., Press, M. C., Callaghan, T. V., Aerts, R., Trosien, F., Riemann, P. et al. (2003) Decomposition of sub-arctic plants with differing nitrogen economies: A functional role for hemiparasites. Ecology, 84, 3209-3221. <https://doi.org/10.1890/02-0426>

Royal Botanical Gardens KEW. (2008) Seed Information Database (SID). Version 7.1. Available: http://data.kew.org/sid/ [Accessed 05/2008].

Royal Botanical Gardens KEW. (2008) Seed Information Database (SID). Version 7.1. Available: http://data.kew.org/sid/ [Accessed 05/2011].

Royal Botanical Gardens KEW. (2008) Seed Information Database (SID). Version 7.1. Available: http://data.kew.org/sid/ [Accessed 05/2014].

Sandel, B., Corbin, J. & Krupa, M. (2011) Using plant functional traits to guide restoration: A case study in California coastal grassland. Ecosphere, 2, 1-16. <https://doi.org/10.1890/Es10-00175.1>

Schroeder‐Georgi, T., Wirth, C., Nadrowski, K., Meyer, S. T., Mommer, L. & Weigelt, A. (2016) From pots to plots: hierarchical trait‐based prediction of plant performance in a mesic grassland. Journal of Ecology, 104, 206-218.

Schweingruber, F. H. & Landolt, W. (2005) The Xylem Database. In: WSL, S. F. R. I. (ed.).

Sheremetev, S. (2005) Herbs on the soil moisture gradient (water relations and the structural-functional organization) (Russian). Moscow: KMK.

Shipley, B. (1995) Structured interspecific determinants of specific leaf area in 34 species of herbaceous angiosperms. Functional Ecology, 9, 312-319. <https://doi.org/10.2307/2390579>

Shipley, B. (2002) Trade-offs between net assimilation rate and specific leaf area in determining relative growth rate: relationship with daily irradiance. Functional Ecology, 16, 682-689. <https://doi.org/10.1046/j.1365-2435.2002.00672.x>

Shipley, B. & Lechowicz, M. J. (2000) The functional co-ordination of leaf morphology, nitrogen concentration, and gas exchange in 40 wetland species. Ecoscience, 7, 183-194.

Shipley, B. & Vu, T. T. (2002) Dry matter content as a measure of dry matter concentration in plants and their parts. New phytologist, 153, 359-364. <https://doi.org/10.1046/j.0028-646X.2001.00320.x>

Siefert, A., Fridley, J. D. & Ritchie, M. E. (2014) Community functional responses to soil and climate at multiple spatial scales: when does intraspecific variation matter? Plos one, 9, e111189.

Smith, S. W., Woodin, S. J., Pakeman, R. J., Johnson, D. & van der Wal, R. (2014) Root traits predict decomposition across a landscape-scale grazing experiment. New phytologist, 203, 851-62. <https://doi.org/10.1111/nph.12845>

Spasojevic, M. J. & Suding, K. N. (2012) Inferring community assembly mechanisms from functional diversity patterns: the importance of multiple assembly processes. Journal of Ecology, 100, 652-661. <https://doi.org/10.1111/j.1365-2745.2011.01945.x>

Takkis, K. (2014) Changes in plant species richness and population performance in response to habitat loss and fragmentation. Dissertationes Biologicae Universitatis Tartuensis, 255.

Thuiller, W. Traits of European Alpine Flora. In: Thuiller, W. (ed.). Centre National de la Recherche Scientifique.

Tribouillois, H., Fort, F., Cruz, P., Charles, R., Flores, O., Garnier, E. & Justes, E. (2015) A functional characterisation of a wide range of cover crop species: Growth and nitrogen acquisition rates, leaf traits and ecological strategies. Plos one, 10, e0122156. <https://doi.org/10.1371/journal.pone.0122156>

Tucker, S. S., Craine, J. M. & Nippert, J. B. (2011) Physiological drought tolerance and the structuring of tallgrass prairie assemblages. Ecosphere, 2, 1-19. <https://doi.org/10.1890/Es11-00023.1>

Vergutz, L., Manzoni, S., Porporato, A., Novais, R. & Jackson, R. (2012) A global database of carbon and nutrient concentrations of green and senesced leaves. Oak Ridge, Tennessee, USA: Oak Ridge National Laboratory Distributed Active Archive Center.

Vile, D. (2005) Significations fonctionnelle et ecologique des traits des especes vegetales: exemple dans une succession post-cultural mediterraneenne et generalisations. PhD, Univ. Montpellier.

Walker, A. P. (2014) A Global Data Set of Leaf Photosynthetic Rates, Leaf N and P, and Specific Leaf Area. Oak Ridge, Tennessee, USA: Oak Ridge National Laboratory Distributed Active Archive Center.

Wang, H., Harrison, S. P., Prentice, I. C., Yang, Y., Bai, F., Furstenau Togashi, H., Wang, M. et al. (2017) The China Plant Trait Database. PANGAEA.

Wirth, C. & Lichstein, J. W. (2009) The imprint of species turnover on old-growth forest carbon balances-Insights from a trait-based model of forest dynamics. In: Wirth, C., Gleixner, G. & Heimann, M. (Eds), Old-Growth Forests: Function, Fate and Value. New York, Berlin, Heidelberg: Springer, 81-113.

Wright, I. J., Reich, P. B., Westoby, M., Ackerly, D. D., Baruch, Z., Bongers, F., Cavender-Bares, J. et al. (2004) The worldwide leaf economics spectrum. Nature, 428, 821-827. <https://doi.org/10.1038/nature02403>

Zheng, W. (1983) Silva Sinica Beijing: China Forestry Publishing House.

unpublished data from:

Abedi, M. Iranian Plant Trait Dataset

Blonder, B. Photosynthesis and Leaf Characteristics Database

Cadotte, M. Herbaceous plants of Rouge National Urban Park

Chapin, F. S. III Tundra Plant Traits Database

Chmurzynski, A. Trait Data from Niwot Ridge LTER (2016)

Forey, E. Plant Coastal Dune Traits (France, Aquitaine)

Harzé, M. Harze Trait Intravar: SLA, LDMC and Plant Height for Calcareous Grassland Species in South Belgium

Lanta, V. Meadow Plant Traits: Biomass Allocation, Rooting depth

Leishman, M. New South Wales Plant Traits Database

Mitchell, R. Traits of Hypochaeris radicata under shade and drought conditions

Read, Q. Maxfield Meadow, Rocky Mountain Biological Laboratory - LMA

Read, Q. Rocky Mountain Biological Laboratory WSR/gradient plant traits

Römermann, C. Herbaceous Plants Traits From Southern Germany

Sheremetev, S. The Global Leaf Traits

Shipley, B. Leaf and Whole Plant Traits Database

Sørensen, M. V. Leaf traits from ECOSHRUB Dovrefjell Norway

Valladares, F. Traits for Common Grasses and Herbs in Spain

Wright, I. Overton/Wright New Zealand Database
