# Supplementary material for: Functional rarity of plants in German hay meadows — Patterns on the species level and mismatches with community species richness
Source: Ecol Evol. 2022 Oct 1;12(10):e9375. doi: 10.1002/ece3.9375 (PMC9526122; doi:10.1002/ece3.9375)
Supplement: Supplementary file 6 — Appendix S4 [file ECE3-12-e9375-s002.docx]

# Appendix S4. Species richness model comparison

To assess the robustness of the observed relationships between species richness and environmental variables, we compared our original species richness model (n = 6500) to a model based on a subset of relevés where size information was available (n = 3237; hereafter referred to as “subset model”). The initial subset model comprised all 12 environmental variables that were used in the original model and relevé area as additional covariate. Fitting and simplification of the boosted regression tree (BRT) model followed the procedure described in the methods section of the main document. A permutation test for Moran’s I statistic was performed to check for spatial autocorrelation in the residuals of the final model within a distance of 43 518 m, so each relevé had at least one neighbor.

As size information was missing for relevés in the north-western part of Germany (especially North Rhine-Westphalia, but also smaller regions across Germany), the subset does not represent a proper spatial sample of the original dataset (Fig. S4-1). Still, it represents the environmental conditions in the original dataset surprisingly well (Fig. S4-2). Differences in the distribution of values can especially be observed for climatic variables (temperature variability, mean annual temperature, annual precipitation; Fig. S4-2), which is likely due to poor spatial sampling in the subset. Explained variance of the subset BRT model was slightly lower compared to the original model (original model: cross-validation correlation mean = 0.750; subset model: cross-validation correlation mean = 0.711) and residuals showed no spatial autocorrelation (Moran’s I = -0.003, p > 0.05).

The subset model contained almost all variables of the final original species richness model (Fig. S4-3 and S4-4). While forest area was excluded, relevé size was included in the subset model. Species richness increased with relevé size up to an area of 100 m² and slightly decreased for larger relevés (Fig. S4-4). Though relative importance of the individual variables differed between the models, relationships between species richness and environmental variables were largely consistent across models (Fig. S4-3 and S4-4). Differences in the relationships could be observed for temperature variability, which showed a stronger positive relationship to species richness in the subset model, and for annual precipitation, which showed a negative relationship in the subset model instead of the hump-shaped relationship observed in the original model (Fig. S4-3 and S4-4). These differences are likely due to poor representation of the data distribution for these variables in the subset, as many observations for low annual precipitation or intermediate and high temperature variability were missing in the subset (Fig. S4-2). Though there is a positive effect of relevé size on species richness, results for the environmental variables are robust across models, suggesting that relevé size should not strongly affect the results and the conclusions of the study.


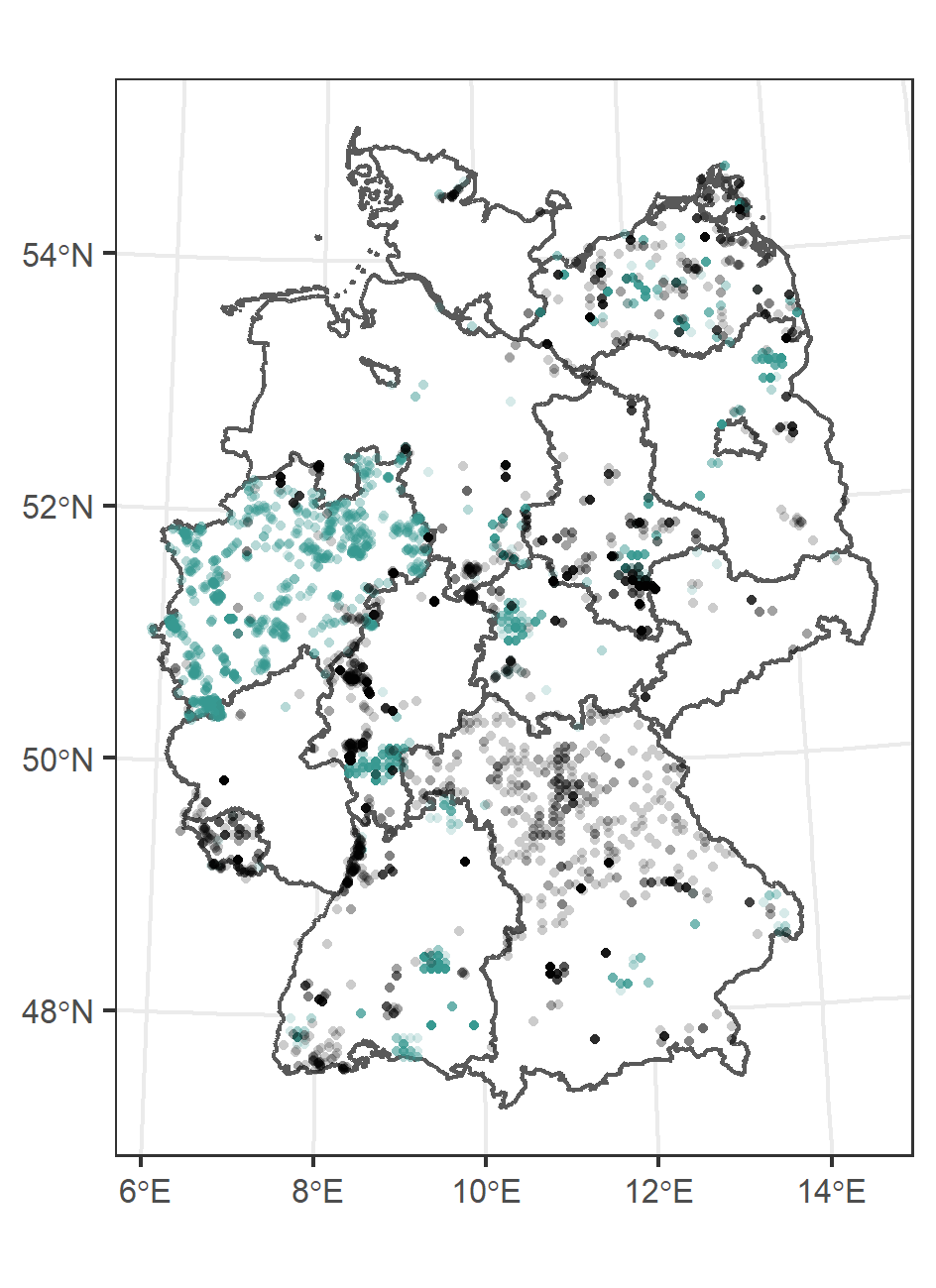


Fig. S4-1 Distribution of 6500 selected hay meadows across Germany that were used in the original boosted regression tree (BRT) model of species richness (all points). Relevés without size information are shown in turquoise. These were omitted in the subset BRT model of species richness. Darker colors indicate locations of higher relevé density.


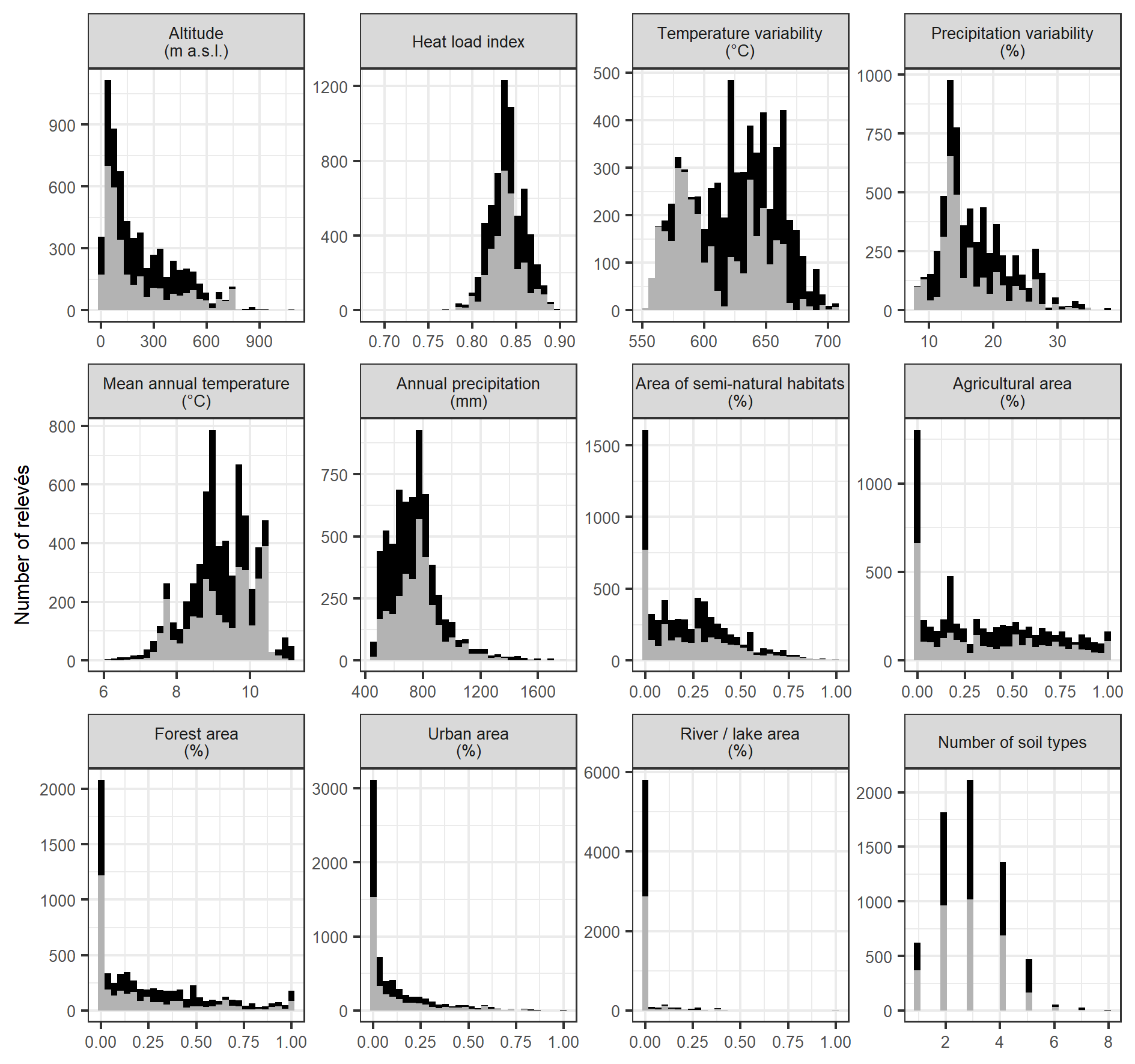


Fig. S4-2 Distribution of the selected relevés across 12 environmental variables included in the initial boosted regression tree (BRT) models for species richness. The distribution of relevés included in the original BRT model is shown in black (n = 6500), while the distribution for the relevés included in the subset BRT model is shown in gray (n = 3237).


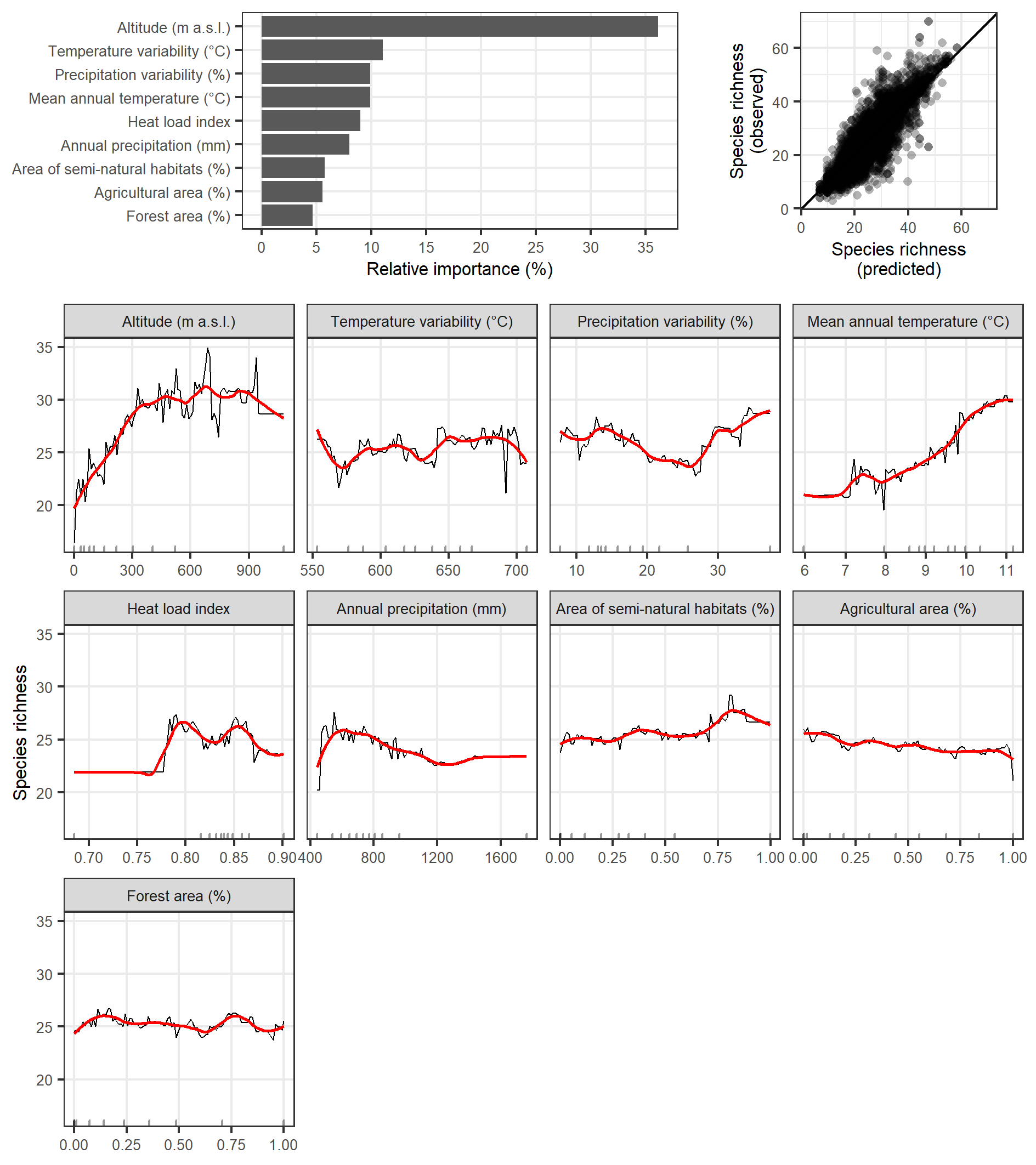


Fig. S4-3 Results from the final boosted regression tree model for species richness per relevé for all relevés included in the original dataset of the study (n = 6500). Upper plot row shows relative importance of the predictive variables included in the final model (left) and relationship between observed and predicted values from the final model (right). Lower plot rows show partial dependence plots for predictive variables included in the final model, ordered by their relative importance (decreasing from top left to bottom right). Black line: fitted relationship between response and predictive variable; red line: smooth representation of fitted relationship; tick marks on x-axes: 10 percent quantiles for values of the predictive variables.


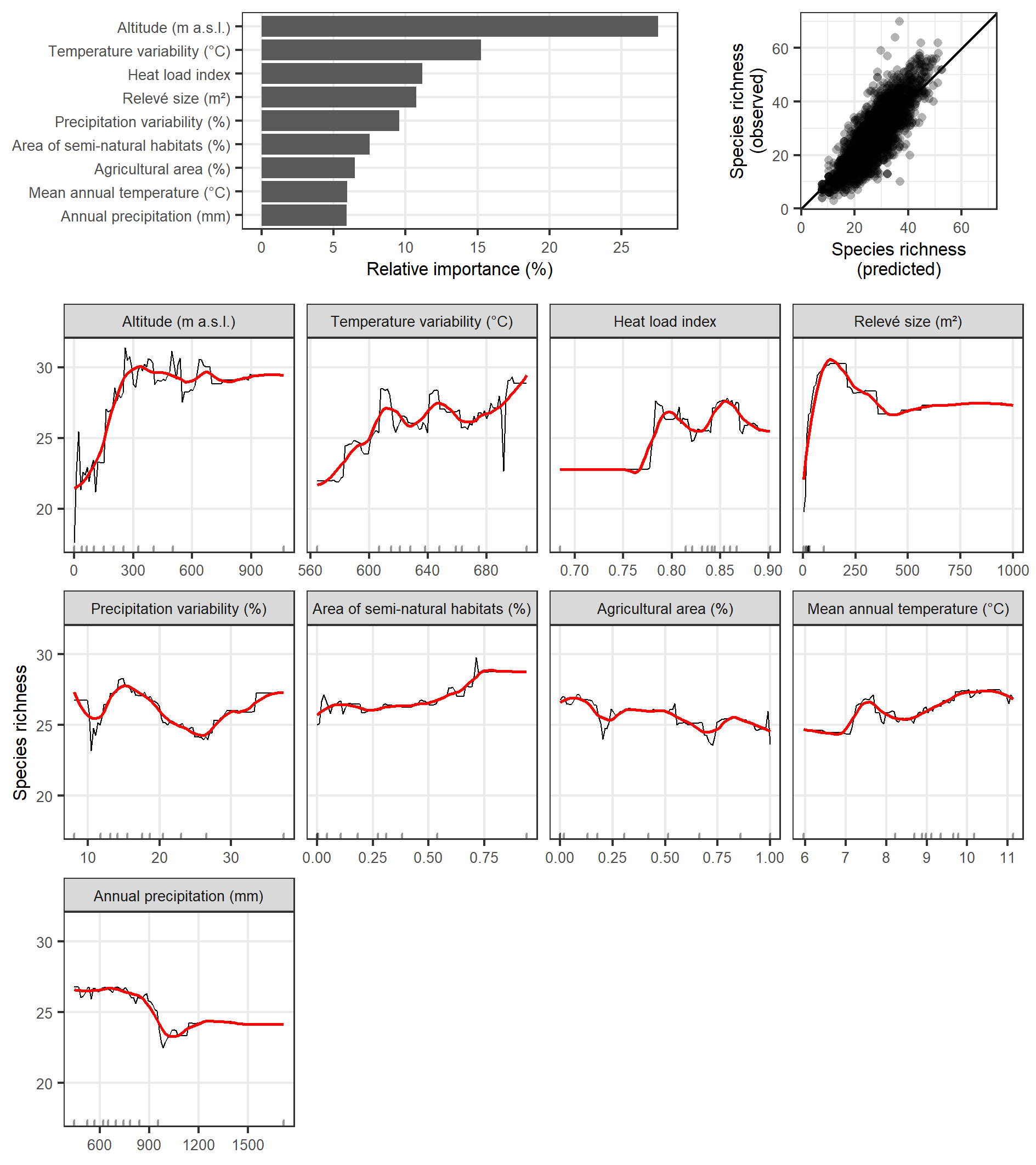


Fig. S4-4 Results from the final boosted regression tree model for species richness per relevé for relevés where size information was available (n = 3237). Upper plot row shows relative importance of the predictive variables included in the final model (left) and relationship between observed and predicted values from the final model (right). Lower plot rows show partial dependence plots for predictive variables included in the final model, ordered by their relative importance (decreasing from top left to bottom right). Black line: fitted relationship between response and predictive variable; red line: smooth representation of fitted relationship; tick marks on x-axes: 10 percent quantiles for values of the predictive variables. Please note that the x-axis of the variable relevé size was set to a maximum of 1000 m² (representing 99% of the data) to ensure readability and consequently does not represent the whole range of values observed for relevé size (maximum relevé size = 8460 m²). However, the pattern does not change for relevés larger than 1000 m².
